# Development and assessment of the outpatient module of the Global-PPS: a standardized approach for measuring outpatient antimicrobial prescribing

**DOI:** 10.1093/jacamr/dlaf115

**Published:** 2025-07-04

**Authors:** Annelies Boven, Ann Versporten, Ines Pauwels, Philip Oshun, Oyinlola Oduyebo, Jimmy Keustermans, Nele Brusselaers, Erika Vlieghe

**Affiliations:** Department of Family Medicine and Population Health, University of Antwerp, Antwerp, Belgium; Department of Family Medicine and Population Health, University of Antwerp, Antwerp, Belgium; Department of Family Medicine and Population Health, University of Antwerp, Antwerp, Belgium; Department of Medical Microbiology and Parasitology, College of Medicine, Lagos University Teaching Hospital, Lagos, Nigeria; Department of Medical Microbiology and Parasitology, College of Medicine, Lagos University Teaching Hospital, Lagos, Nigeria; Department of Family Medicine and Population Health, University of Antwerp, Antwerp, Belgium; Department of Family Medicine and Population Health, University of Antwerp, Antwerp, Belgium; Department of Women’s and Children’s Health, Karolinska Institutet, Stockholm, Sweden; Department of Public Health & Primary Care, Ghent University, Ghent, Belgium; Department of Family Medicine and Population Health, University of Antwerp, Antwerp, Belgium; Department of General Internal Medicine, Infectious Diseases and Tropical Medicine, University Hospital Antwerp, Antwerp, Belgium

## Abstract

**Objectives:**

Many tools for antimicrobial use surveillance have been exclusively developed for inpatient settings, although >80% of all antimicrobials are prescribed in outpatient care. In this study, we describe the methodology and first evaluation of the outpatient Global Point Prevalence Survey (Global-PPS).

**Methods:**

Global-PPS developed a standardized tool to measure outpatient antimicrobial prescribing patterns. A core set of variables was collected for all ambulatory patients, and more detailed data for those prescribed antimicrobials during the survey. A questionnaire containing 34 questions on experiences with the outpatient Global-PPS was administered to users who had used the outpatient module of the Global-PPS between May 2023 and December 2024.

**Results:**

In total, 39 (29.1%) participants of the outpatient Global-PPS from 25 (71.4%) different healthcare facilities responded to the questionnaire, of which 25 (from 16 facilities) completed it. Most respondents were from Nigeria (50%), Ghana (12.5%) or Guinea (10%).

Respondent satisfaction was very high (>90%, *n* = 25), although additional training and support tools seemed to be needed. Most facilities (75%, *n* = 16) encountered certain barriers to conducting the survey, including challenges in obtaining patient information during consultation (44%) or accessing medical records (28%), and a lack of trained staff (16%).

**Conclusions:**

The outpatient Global-PPS is the first freely available web-based standardized tool for measuring antimicrobial use in outpatient care worldwide, featuring an online data entry platform, validation and real-time reporting. This study confirmed a high degree of satisfaction among participants, although additional support is needed to train staff and overcome certain barriers, including access to patient information.

## Introduction

Global antimicrobial stewardship efforts have primarily focused on inpatient settings, since antimicrobial use surveillance and stewardship are often more feasible in controlled and confined hospital settings than in the more heterogeneous and resource-limited outpatient setting. Although most antibiotics are prescribed in outpatient settings,^1^ many currently available tools and methodologies for the surveillance of (global) antimicrobial use are mostly concentrated on inpatient care.^2–4^

Previous research has addressed the growing need for feasible and valid tools to survey antimicrobial use and prescription quality in outpatient settings.^5–7^ In the outpatient sector, several metrics for antimicrobial use surveillance have been identified, for example DDDs per 1000 inhabitants per day (DID), prescriptions or treatments per 1000 population (antibiotic prescribing rate; APR) or per defined number of physician contacts,^6,8,9^ Days of therapy (DOT) per 1000 inhabitants per day (DOTID), and the proportion of prescriptions containing an antimicrobial.^9^ These metrics, however, contain merged results for certain antimicrobial subgroups, which reduces the granularity and could mask important differences in antimicrobial prescribing patterns.

Repeated surveys can monitor antimicrobial prescribing patterns and provide more granularity. These methods, mainly available in high-income countries (HICs), often utilize electronic medical records to facilitate data collection and analysis,^10,11^ although their use is more challenging when the data are of insufficient quality, or not collected and stored in a standardized way.^10^ These limitations make the use of electronic medical records infeasible in many settings without high-quality electronic file systems.

Many antimicrobial use surveillance tools are poorly adapted to the unique challenges of low- and middle-income countries (LMICs).^12^ The Global Point Prevalence Survey for Antimicrobial Consumption (Global-PPS) is among the first web-based applications offering a standardized methodology for antimicrobial use surveillance, suitable for both LMIC and HIC settings. The Global-PPS methodology and tool have been freely available for inpatient care since 2014,^13^ and have supported the surveillance of over 575 000 patients across at least 1150 hospitals from 97 countries (https://www.global-pps.com/timeline/). The Global-PPS outpatient module was developed following the inpatient counterpart, to meet the need for regular antimicrobial use monitoring in outpatient care.

This study aimed to outline the methodology and development of the outpatient Global-PPS module, evaluate implementation feasibility through participant feedback, and highlight key lessons learned.

## Methods

### Study design

The extension of the already existing Global-PPS with an outpatient module was based on the Global-PPS inpatient module.^14^ The new outpatient module was designed to evaluate antimicrobial prescribing among all outpatients, defined as patients receiving care without overnight admission, across diverse healthcare settings including hospitals, outpatient clinics and primary healthcare facilities.

The development of the survey protocol, in early 2022, went through multiple iterations, with thorough cross-checking by various Global-PPS participants who were already familiar with the inpatient protocol (https://www.global-pps.com/acknowledgements/). From September to December 2022, the protocol was piloted in over 20 Nigerian outpatient units for a total of over 500 outpatients. In May 2023, the outpatient module was released for public use, and improvements have been continuously added based on participant feedback (Appendix S2, available as Supplementary data at *JAC-AMR* Online).

The methodology featured three key differences from the inpatient Global-PPS module (Table S1).

The first involved collecting baseline variables for all eligible patients, including age group, sex, admission status and basic diagnostic information (symptoms and diagnostic test ordered), to enable in-depth analysis of prescribing patterns by age and patient characteristics (Table 1). More detailed variables on patient characteristics, antimicrobial use and quality indicators were collected solely for patients with an active antimicrobial, acting as numerators (Table 1).

**Table 1.** Rationale for the method and collected variables in the outpatient Global-PPS modules

Element	Details	Rationale
Inclusion criteria	Patients attending the outpatient setting and not staying overnight, with the exception of emergency or observation wards.	Patients in emergency or observation wards are captured in the outpatient module, irrespective of admission status to allow capture of the most appropriate (complete) denominator.
Denominators	All outpatients seen during the survey. For them, baseline variables are collected.	Enable the comparison of different age groups and patient characteristics.
Numerator	All outpatients seen during the survey with an antimicrobial prescription. For them, more detailed data are collected.	Obtain detailed insights in antimicrobial prescribing patterns.
Duration of survey	Short period of at least half a day (4 h).	Patients seen in outpatient care are not all present at the same time in the facility, hence data collection covers a predefined period.
Applicable settings	Outpatient & emergency wards Outpatient clinics Dental clinics Day surgery units Primary healthcare centres GP and Family Medicine practices	All healthcare institutions where patients can receive outpatient care. It captures a large variety of outpatient settings.

Variables	Collected for	Rationale
Age group and sex	All patients	To report antimicrobial prescribing results by patient group.
Symptoms/ordered test		Collection of diagnostic information leading to diagnosis setting. It is aimed to group a set of symptoms together with an ordered test, to better understand to whom and in what setting antimicrobials are or are not prescribed.
Admission status	All patients seen in emergency/observation wards	To account for patients who (likely) will be or are admitted during or after the survey.
Diagnostic tests (biomarkers, point-of-care tests and/or rapid diagnostic tests)	Patients receiving an antimicrobial prescription	As a quality indicator for antimicrobial prescribing.
Cultures taken before the start of the antimicrobial		As a quality indicator for antimicrobial prescribing, aimed at settings with sufficient diagnostic capacity.
Penicillin allergy		To evaluate the rationale behind the choice for the antimicrobial prescription.
Comorbidities		To collect information on risk factors for antimicrobial prescribing.
Details on the antimicrobial treatment (name, dose, route of administration, intended duration, new/ongoing/switched drug)	Patients receiving an antimicrobial prescription. Multiple antimicrobials possible per patient.	To calculate the prevalence of antimicrobials, and use this information for guideline adherence.
Clinical diagnosis and infection origin (CAI, HAI, prophylaxis)	Patients receiving an antimicrobial prescription	To evaluate the rationale behind the choice for the antimicrobial prescription
Guideline availability and compliance (for drug, dose, route of administration and intended duration)		As a quality indicator for antimicrobial prescribing.
Reason in notes		As a quality indicator for antimicrobial prescribing.

CAI, community-acquired infection; HAI, hospital-acquired infection.

Secondly, the module requires surveillance for at least 4 h on one or more representative days (excluding weekends and bank holidays), providing flexibility to accommodate differences in patient turnover. Sampling of patients was not allowed.

Thirdly, both prospective (during consultation) and retrospective data collection were allowed, the latter only if all necessary information could be retrieved from medical files.

In 2023, the web-based Global-PPS application for data entry and validation was expanded to include the outpatient protocol. All participating facilities remained the owner of their database. Entered data were stored at the secure servers of the University of Antwerp.

Further details on data collection, as well as the data collection forms and protocol, are available in Appendices S1–S3.

### Evaluation of outpatient Global-PPS

A questionnaire was developed based on a similar (unpublished) survey for the inpatient module of the Global-PPS (Table S2). Responses were gathered using the web-based SurveyMonkey tool,^15^ targeting all registered users who had participated with their facility/network in the outpatient Global-PPS between May 2023 (release of the outpatient Global-PPS) and December 2024.

The questionnaire included 34 questions on participants’ perception and experience with conducting the outpatient Global-PPS, their opinion and any suggestions for the current methodology and support tools. Respondent satisfaction with different parts of the tool was measured using a 5-point Likert scale ranging from ‘not satisfied at all’ to ‘extremely satisfied’.

After in-house piloting of the full questionnaire by four members of the research team and one participant, the questionnaire was slightly adapted to enhance the clarity and feasibility, and was distributed in early October 2024, in English only. Three reminders were sent after the initial release, supplemented with a French translation, and an extra reminder to complete the full survey was sent at the end of the study.

Respondents were informed about this study at the beginning of the questionnaire. Their participation counted as informed consent.

Differences in responses between participants from the same facility in the facility-related questions (e.g. facility type, number of surveyed days and units etc.), were approached by taking the mean for continuous variables, and the median for categorical variables. For the latter, if responses were evenly split at 50%, we selected either the highest number (for the number of involved staff) or selected ‘presence’ for the presence or absence of certain requirements or barriers. In the case of duplicate entries from the same respondent, the most comprehensive entry was retained. All facility names and e-mail addresses were pseudonymized before data analysis and descriptive analyses were conducted in Microsoft Excel and R (version 4.3.1). No medical or personally identifiable data were collected apart from the e-mail address if respondents wished to be acknowledged.

## Results

The questionnaire was sent to 134 users belonging to 34 facilities or networks.

In total, 40 participants from 26 different healthcare facilities initiated the questionnaire, of which 25 (from 16 facilities/networks) fully completed it. Two responses were excluded: one was a duplicate entry and the other had >90% missing entries. One respondent was excluded from the response rate calculations only as they remained completely anonymous and listed a country of origin inconsistent with the registered users.

The total response rate was 29.1% (*n* = 39) among respondents and 71.4% (*n* = 25) among facilities/networks, when adjusting for multiple users per facility/network. Details on the geographical localization, type of facility, self-reported position and time investment are available in Table 2 (see Appendix S3 for definition of the facility types).

**Table 2.** Characteristics of the respondents and the facilities who participated in the evaluation questionnaire as part of the outpatient Global-PPS

Questionnaire	Participants	Facilities^a^
Number of responses, *n* (%)	39 (29.1)	25 (71.4)
Number of completed responses, *n* (%)	25 (62.5)	16 (61.5)
Region, *n* (%)		
Continent: Africa	33 (84.6)	19 (76.0)
Nigeria	20 (51.3)	10 (40.0)
Ghana	5 (12.8)	1 (4.0)
Guinea	4 (10.3)	4 (16.0)
South Africa	2 (5.1)	2 (8.0)
Burkina Faso	1 (2.6)	1 (4.0)
Kenya	1 (2.6)	1 (4.0)
Continent: Other (Asia, Europe, North America)	5 (12.8)	5 (20.0)
Belgium	1 (2.6)	1 (4.0)
Canada	1 (2.6)	1 (4.0)
Georgia	1 (2.6)	1 (4.0)
Germany	1 (2.6)	1 (4.0)
Pakistan	1 (2.6)	1 (4.0)
Unknown	1 (2.6)	1 (4.0)
Type of facility, *n* (%)		
Tertiary or specialized hospital	16 (41.0)	10 (40.0)
Primary or secondary hospital	14 (35.9)	6 (24.0)
Outpatient clinic or primary healthcare facility	9 (23.1)	9 (36.0)
Self-reported positions, *n* (%)		
Microbiologist	9 (23.1)	—
Infectious diseases specialist	6 (15.4)	—
Clinician with other specialty	4 (10.3)	—
Pharmacist	10 (25.6)	—
Nurse	3 (7.7)	—
Administrative staff	1 (2.6)	—
Medical student	4 (10.3)	—
Other	2 (5.1)	—
Data collection method, *n* (%)		
On paper/in electronic files	33 (86.2)	20 (80.0)
Directly into the online data entry tool	5 (12.8)	5 (20.0)
Need for ethical/institutional approval, *n* (%)		
Any form of institutional approval	31 (79.5)	20 (80.0)
Ethical clearance	14 (35.9)	7 (28.0)
Informed consent	7 (17.9)	6 (24.0)
Time investment in data collection, median (IQR)		
Data collection time (min) for one patient without any antimicrobial	5 (2–10)	7.5 (3–10.3)
Data collection time (min) for one patient on an antimicrobial	10 (7.8–15)	10 (9.1–16)
Time investment in data entry, median (IQR)		
Data entry time (min) for one patient without any antimicrobial	4.5 (2–5.3)	5 (2.5–8.3)
Data entry time (min) for one patient on an antimicrobial	10 (5–10)	10 (5–10)
Total number of patients by survey, *n* (%)		
<50	7 (21.9)	6 (28.6)
51–100	7 (21.9)	4 (19.0)
101–200	6 (18.7)	4 (19.0)
201–500	7 (21.9)	5 (23.8)
>500	5 (15.6)	2 (9.5)
Materials used, *n* (%)		
Personalized feedback for single facility	14 (56.0)	7 (43.8)
Personalized merged feedback for multiple facilities	5 (20)	3 (18.8)
Raw data export	13 (52.0)	9 (56.3)
None yet, but planning to	5 (20.0)	5 (31.3)

^a^Answers from respondents were merged at the facility level by taking the mean or median response. If responses were evenly split at 50%, we took the highest number or ‘presence’ for binary variables.

### Organization at facility level

Over half of the facilities had conducted the survey for at least 3 days (60%, *n* = 15) among a median of 4 units (IQR: 2–9). Many facilities required institutional approval (80%, *n* = 20) (Table 2). A few needed ethical approval (28%, *n* = 7) and/or informed consent (24%, *n* = 6) (Table 2). Informed consent was asked by verbal (83%, *n* = 5) or written agreement (16.7%, *n* = 1).

Among 20 facilities that reported which staff were involved in the PPS, most facilities mentioned at least one infectious disease specialist (65%, *n* = 13), clinician with other specialty (65%, *n* = 13), pharmacist (60%, *n* = 12), nurse (60%, *n* = 12), administrative staff (45%, *n* = 9), medical student (30%, *n* = 6), nursing student (10%, *n* = 2) or other staff (55%, *n* = 11) in the organization, the data collection and/or data entry of the outpatient Global-PPS.

Most respondents (69.2%, *n* = 27) would prefer to survey their facility at least every 3–6 months.

### Time investment

Most facilities had collected data on paper forms (76%) or in an electronic file (4%) before data entry, whereas 20% entered data directly into the website (Table 2).

For a single patient, respondents estimated a median time for data collection of 5 min (IQR: 2–10, *n* = 29) among patients without any antimicrobial prescription during the survey, and 10 min (IQR: 7.8–15, *n* = 29) among patients with an antimicrobial prescription (Table 2).

The median estimated time that respondents spent on data entry for a single patient was 4.5 min (IQR: 2–5.3, *n* = 29) among patients without any antimicrobial prescription during the survey, and 10 min (IQR: 5–10, *n* = 29) among patients with an antimicrobial prescription (Table 2).

### Evaluation of the methodology

Overall, most respondents (79.3%) considered the outpatient Global-PPS methodology sufficiently detailed (Table 3). The most common suggestions included adding extra options for presenting symptoms, diagnostic tests or comorbidities.

**Table 3.** Evaluation of the outpatient Global-PPS and encountered barriers by respondents and facilities

	Participants, *n* (%)			Facilities, *n* (%)		
	Not/somewhat satisfied	Very/extremely satisfied	Did not use it/NA	Not/somewhat satisfied	Very/extremely satisfied	Did not use it/NA
Protocol	1 (3.3)	28 (93.3)	1 (3.3)	0 (0.0)	19 (100.0)	0 (0.0)
Collection forms	3 (10.0)	27 (90.0)	0 (0.0)	1 (5.3)	18 (94.7)	0 (0.0)
Data entry tool	2 (6.7)	26 (86.7)	2 (6.7)	2 (10.5)	16 (84.2)	1 (5.3)
Feedback report	6 (20.0)	18 (60.0)	6 (20.0)	4 (21.1)	14 (73.7)	1 (5.3)
User manual	6 (20.0)	19 (63.3)	5 (16.7)	4 (21.1)	13 (68.4)	2 (10.5)
Tutorial videos	8 (26.7)	13 (43.3)	9 (30.0)	6 (31.6)	8 (42.1)	5 (26.3)
Helpdesk	7 (23.3)	13 (43.3)	10 (33.3)	5 (26.3)	10 (52.6)	4 (21.1)
Promotional materials	9 (30.0)	11 (36.7)	10 (33.3)	6 (31.6)	7 (36.8)	6 (31.6)
Feedback on protocol, *n* (%)						
Sufficient detail	23 (79.3)			14 (77.8)		
Not enough detail	4 (13.8)			2 (11.1)		
Too much detail	0 (0.0)			0 (0.0)		
Sometimes not enough, sometimes too much detail	2 (6.9)			2 (11.1)		
Impact of Global-PPS on understanding of prescribing patterns in facility						
Number of responses	25			16		
Score 0 (no impact at all)—100 (very high impact), mean (SD)	77 (±23)			80 (±26)		

Answers from respondents were merged at the facility level by taking the mean or median response.

Most respondents who had used the Global-PPS outpatient method and/or its support tools, were very or extremely satisfied with the protocol (97%), data collection forms (90%) and data entry tool (93%), although the degree of satisfaction was lower for the user manual (76%), feedback report (75%), helpdesk (65%), tutorial videos (62%) and promotional materials (55%) (Table 3).

### Barriers

**Figure 1.**
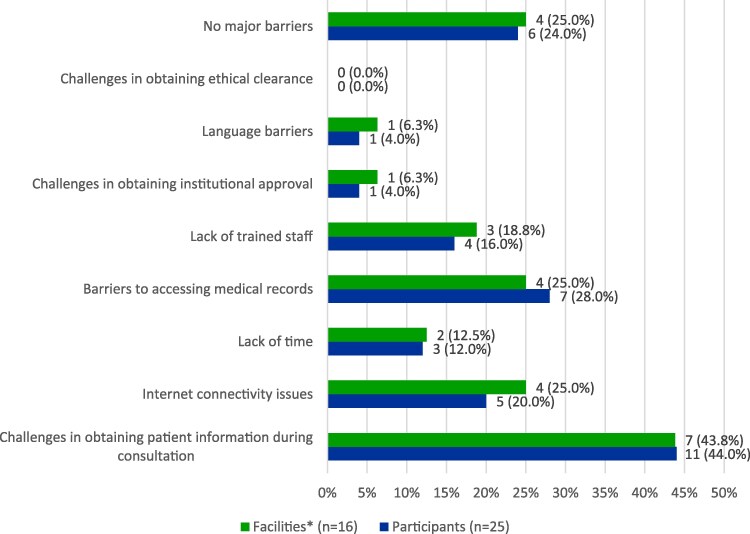
Common barriers encountered by participants and facilities when conducting the outpatient module of the Global-PPS. *Answers from respondents were merged at the facility level by taking the median response. In the case of responses being evenly split at 50%, the barrier was considered present in that facility. Participants and facilities could select multiple responses.

The vast majority of respondents (76%, *n* = 19) and facilities (75%, *n* = 12) experienced barriers to conducting the outpatient Global-PPS (Figure 1). These included challenges in obtaining patient information during the consultation (44%, *n* = 11), barriers in accessing medical records (28%, *n* = 7) and lack of trained staff (16%, *n* = 4). In contrast, problems with obtaining ethical clearance and institutional approval were rarely observed (Figure 1).

Many respondents who gave additional suggestions or feedback recommended providing additional training opportunities (42%, *n* = 5). A few respondents suggested financial support to compensate staff time, and more structural support to improve access to patient information obtained from medical records or during consultation.

## Discussion

The outpatient Global-PPS offers the first standardized web-based tool for measuring antimicrobial prescribing patterns across a wide range of infectious diseases in diverse outpatient settings worldwide. We believe this new module fills an important gap in outpatient antimicrobial stewardship, where targeted strategies to monitor the high burden of antimicrobial use have been lacking.

This study presents the first findings from an evaluation of this new module, demonstrating that the survey protocol, data collection forms and online tool were perceived as satisfactory by >90% of the participants, although opinions on the other support tools were more divided.

In line with the CDC core elements of outpatient stewardship, i.e. ‘commitment, action for policy and practice, tracking and reporting, and education and expertise’,^16,17^ this tool provides a platform that enables tracking and reporting antimicrobial use, by facilitating data collection on (facility-level) antimicrobial prescribing patterns and comparison with a regional benchmark.

### Organization of the outpatient Global-PPS

This study brought several insights into organizing an antimicrobial use surveillance study like Global-PPS among outpatient facilities, including the frequent need for institutional and ethical approval, and occasionally informed consent. Obtaining approval was the responsibility of each participating facility, which was seldom an issue.

Often, multidisciplinary teams were organized, mostly consisting of clinicians with varying specialties, pharmacists, administrative staff and students. Previous studies that made recommendations for outpatient stewardship have emphasized the importance of working with multidisciplinary teams in outpatient stewardship efforts (including antimicrobial use monitoring),^18,19^ even including non-medical professionals in the community, although at least one team member should have sufficient knowledge and expertise on infectious diseases.^19^ Most of these recommendations are, however, primarily focused on the USA.

Among most facilities, the survey was conducted in multiple units for at least 3 days. There seems no consensus on the duration of data collection for point prevalence surveys (PPSs) on antimicrobial use in outpatient care, since some collect data for one day,^20^ and others for multiple days.^21,22^ The protocol for the outpatient Global-PPS does not specify a minimum survey time or sample size, allowing for flexibility but potentially affecting consistency of the data. Establishing evidence-based recommendations for the survey time and sample size could improve the robustness of this surveillance tool.

Data collection was mostly conducted on paper forms, although a few facilities opted for direct entry in the tool. The frequent use of paper forms was expected, given the high participation degree among LMICs. Large variations between (the availability of) IT infrastructures of participating facilities mandate the need for both offline data collection as well as direct data entry. For instance, a study from Uganda comparing the (inpatient) Global-PPS with the WHO PPS addressed the need for better accommodation of the Global-PPS in resource-limited settings where the IT infrastructure is inadequate and internet connectivity is unreliable,^12^ which has been addressed meanwhile by offering an import functionality. On the other hand, a large systematic review on antimicrobial stewardship in outpatient care including 50 studies mostly from the USA, Canada or the UK highlighted the importance of computer-based, accessible programmes to enhance user-friendliness and reduce effort.^23^

The focus on global settings requires adaptation to both resource-limited and resource-rich settings. Some factors for antimicrobial use surveillance are important everywhere, such as human resources and time investment.^24,25^ It seems that data collection and entry for the outpatient Global-PPS are time- and labour-intensive, considering the median estimated time to complete both data collection and entry for one patient was 9.5 min without antimicrobial treatment and 20 min with treatment, although there was much variability in these estimates between respondents. Further research is needed to provide a more precise and unbiased assessment of the time investment, by including a larger number of respondents and accounting for covariate factors such as training and access to patient information from the medical files or during consultation, which were perceived as major barriers in this study.

This workload could hinder prospective surveillance in primary care settings of many countries, where these consultations only take approximately 5 min.^26^ This can increase staff workload, although increased monitoring of antimicrobial consumption can help reduce the burden of antimicrobial resistance and ultimately the burden on healthcare. Despite the lack of comparable outpatient tools, similar high workloads have been previously observed in PPSs of inpatient antimicrobial prescribing,^27–29^ with an estimated mean time per patient ranging from 5 min for data collection only,^30^ to 15 min for data collection and entry.^28,29^ This high workload is likely influenced by a learning curve: repeated participation can accelerate data collection and entry.

The practical implementation of the outpatient module and best practices for conducting this survey warrant further study. Nevertheless, these findings stress the importance of sufficient support for antimicrobial use monitoring and emphasize the demand for repeated training and optimizing antimicrobial use surveillance tools. Therefore, future work of the Global-PPS will aim to provide sufficient training opportunities and explore the potential for electronic medical record integration, especially in HICs.

### Assessment of the outpatient Global-PPS

Most respondents were very satisfied with the outpatient Global-PPS methodology, tool and level of detail, although non-response and social desirability biases could lead to skewed results. However, satisfaction with support resources such as the user manual and feedback report were more divided. Future work could focus on improving these resources, by simplifying and translating the manuals, to overcome execution challenges and increase the efficiency of data collection and entry.

Many respondents additionally reported that the study positively impacted their understanding of the facility’s antimicrobial prescribing patterns, even though only half of them had used the reports, possibly due to the short availability of the automatic feedback reports before the questionnaire. Earlier research has demonstrated the positive effect of individualized feedback on antimicrobial prescribing in outpatient care,^31–33^ underscoring the added value of the personalized feedback reports in the Global-PPS.

Nevertheless, the vast majority of the respondents encountered important barriers while conducting the study, such as challenges in obtaining patient information and accessing medical records, and a shortage of trained staff. Similar barriers have been described for antimicrobial use surveillance and stewardship in outpatient care in the USA, including a lack of recorded indications for antimicrobial prescribing in ambulatory clinics^34^ and a lack of time, financial and administrative support for stewardship activities among paediatric institutions.^35^ Further in-depth research across various outpatient settings is needed to explore the feasibility, identify best practices, such time allocation and institutional support, and uncover potential barriers to antimicrobial use surveillance. In addition, more research is needed on effective data collection methods like ‘observer in cabinet’, exit surveys and immediate file reviews.

To help address these challenges, respondents suggested additional training and financial support, while qualitative research could reveal deeper implementation challenges and inform tailored solutions. Increasing training efforts and providing targeted support will be essential to support participants and increase the survey’s feasibility.

### Strengths and limitations

An important strength of this study was that it was a first evaluation of a novel module, assessed by a wide variety of users across continents and settings. Moreover, we explored organizational and implementation aspects related to conducting this outpatient PPS, such as involved staff and time-investment, which could contribute to estimations of the effort and hence the feasibility of conducting a PPS on antimicrobial use in outpatient settings.

Another strength of this study was the outreach to all participants of the outpatient Global-PPS. The survey was limited to participants from facilities who had actively entered patient data and therefore had experienced the entire process.

Moreover, the inclusion of open questions and opportunities for participants to provide feedback in their own words could address the strengths and weaknesses of this methodology that had not been recognized earlier. Additionally, the questionnaire was translated to French, since many facilities that participated originated from French-speaking countries and regions.

A limitation of this study is the low response rate, limiting the representativeness of the results of our evaluation questionnaire. Nevertheless, this low response was expected since many users are healthcare workers, with generally lower response rates to surveys,^36^ related to their busier schedules. We adjusted for multiple participants per facility/network, because often only a few users have experienced both data collection and entry, resulting in an adjusted response rate of >70%. Furthermore, non-response bias might occur in this study, since respondents with a poorer understanding of English or French, or limited access to their e-mail, likely could not complete the questionnaire. This could direct the results towards a higher degree of satisfaction, fewer reported barriers and potentially more homogeneous results. We tried to mitigate this type of bias by sending regular reminders and including all participants who had ever used this tool until 1.5 years after its first release.

Geographical bias may have influenced study results, considering most respondents were from (West) Africa, particularly Nigeria, Ghana and Guinea. While this distribution is reasonably representative of the origin of participants in the outpatient Global-PPS, most of whom are from (West) Africa (see https://www.global-pps.com/project/outpatient-module/), other regions and HICs are underrepresented. Therefore, our findings may have limited generalizability to other geographical regions and HICs.

In addition, this study might be prone to early adopter bias; facilities that participated early in the outpatient Global-PPS might have better resources to conduct the survey or might be more driven towards antimicrobial stewardship, likely providing more positive feedback than respondents from facilities with fewer resources. However, several facilities from Nigeria and Burkina Faso received funding to conduct this method as part of a quality improvement study, hence might represent more typical facilities from those countries.

Social desirability bias may have further influenced the results towards a more positive outcome, especially since respondents were asked to provide their e-mail address if they wished to be acknowledged in this paper. We tried to minimize this type of bias by providing the possibility to respond completely anonymously and using as much neutral wording as possible. Moreover, many participants have previously participated in the inpatient Global-PPS and might be familiar with the frequent feedback opportunities, which could diminish the effect of social desirability bias. We also tried to limit the sensitive information asked in the survey. Negative response bias may, on the other hand, have played a role when respondents were frustrated with certain aspects of the tool, though this effect was reduced by asking follow-up questions about their experience.

Future research along with objective analyses of the data and results obtained can further explore experiences with outpatient antimicrobial use surveillance methods across countries, healthcare types and ward specialties. Given the heterogeneity of outpatient settings, it is essential to consider stakeholders’ experiences from a wide variety of settings to investigate their needs and provide tailored support.

### Conclusions

The Global-PPS outpatient module was developed in response to the high burden of antimicrobial use in outpatient care and the need for regular antimicrobial prescription monitoring. Results from this first evaluation highlight the feasibility of the outpatient Global-PPS as a standardized tool for measuring antimicrobial use among its first participants. Nevertheless, continuous additional support is necessary to overcome barriers such as challenges in obtaining patient information and shortage of trained staff, for instance by providing dedicated staff training and time compensation, to ultimately minimize participant effort and increase survey feasibility. More experience and validation across continents and settings is needed for a deeper understanding of its applicability and for adapting the tool to various contexts.

## Supplementary Material

dlaf115_Supplementary_Data

## Data Availability

Deidentified data, excluding any personal comments and answers from participants, will be made available upon reasonable request.

## Supplementary data

Tables S1 and S2 and Appendices S1 to S3 are available as Supplementary data at *JAC-AMR*  Online.
