# Supplementary material for: Development and assessment of the outpatient module of the Global-PPS: a standardized approach for measuring outpatient antimicrobial prescribing
Source: JAC Antimicrob Resist. 2025 Jul 4;7(4):dlaf115. doi: 10.1093/jacamr/dlaf115 (PMC12231535; doi:10.1093/jacamr/dlaf115)
Supplement: dlaf115_Supplementary_Data [file dlaf115_supplementary_data.docx]

**Supplementary Materials**

- [**Supplementary Table 1**: Differences in the methodology and collected variables between the out- and inpatient Global-PPS modules.](#SupplementaryTable1)
- [**Supplementary Table 2**: Outpatient Global-PPS evaluation questionnaire](#SupplementaryTable2)
- [**Appendix 1:** Detailed explanation of the data collection for the outpatient Global-PPS](#Appendix1)
- [**Appendix 2**: Data collection forms for the outpatient module of the Global-PPS](#Appendix2)
- [**Appendix 3:** Protocol for the outpatient module of the Global-PPS](#Appendix3)

**Supplementary Table 1**: Differences in the methodology and collected variables between the out- and inpatient Global-PPS modules

|  | Outpatient Global-PPS | Inpatient Global-PPS (with and without HAI-module) |
| --- | --- | --- |
| Denominators | Baseline variables for all patients | The total number of beds available in the ward, along with the total number of inpatients who stayed overnight and were present at 8 AM on the day of the PPS, are recorded. For the HAI module, additional denominators are collected, including the count of patients with an invasive device. |
| Duration of survey | Short period of at least half a day | One point in time, 8 a.m. |
| Applicable settings | Outpatient & emergency wards of acute care or specialised hospitals, outpatient clinics, dental clinics, day surgery units, community & primary healthcare centers, long term care facilities, general practitioner practices | Wards admitting inpatients from primary, secondary, tertiary and specialized care hospitals including paediatric, psychiatric and rehabilitation centres. |
| Inclusion criteria | Patients with <24 hour stay, or in emergency or observation wards | Patients with overnight or >24-hour admission |

**Supplementary Table 2:** Outpatient Global-PPS evaluation questionnaire

| **Questions** | **Answer options** | | | | | **Logic** |
| --- | --- | --- | --- | --- | --- | --- |
| ***Section 1: Introduction*** |  | | | | |  |
| **Q1.* For which country did you collect Global-PPS data?*** | [All available countries in Global-PPS] | | | | | - |
| **Q2.* Please fill in the name of the healthcare facility for which you collected data***  ***We use this data only to correct for multiple responses per facility (i.e. duplicate answers). We will not use your data entered in the Global-PPS outpatient module.*** | [open question] | | | | |  |
| **Q3.* What kind of facility did you collect the results for?** | Outpatient clinic | | | | | - |
|  | Primary healthcare center (for outpatients) | | | | |  |
|  | Primary care hospital | | | | |  |
|  | Secondary hospital | | | | |  |
|  | Tertiary hospital | | | | |  |
|  | Pediatric hospital | | | | |  |
|  | Infectious diseases hospital | | | | |  |
|  | Other specialized hospital | | | | |  |
|  | Other, please specify: | | | | |  |
| **Q4.* What is your main role in the hospital/healthcare facility?** | Microbiology | | | | | - |
|  | Infectious diseases | | | | |  |
|  | Pharmacist | | | | |  |
|  | Other clinician | | | | |  |
|  | Hospital hiegienist | | | | |  |
|  | Nurse | | | | |  |
|  | Medical student | | | | |  |
|  | Nursing student | | | | |  |
|  | Administrative staff | | | | |  |
|  | Other, please specify: | | | | |  |
| **Q5. Please fill in your email address if you would like to be acknowledged in the peer-reviewed paper** | [open question] | | | | | - |
| ***Section 2: Organization at facility level*** |  | | | | |  |
| **Q6. For how many days did you survey your facility?** | Half a day (+- 4hours) | | | 1 day | | - |
|  | 2 days | | | 3 days | |  |
|  | Other, please specify: | | | >3 days | |  |
| **Q7. How many units/clinics/services did you survey?** | Options: 1-25; Other, please specify: … | | | | | - |
| **Q8. How often would you like to conduct the survey?** | Every 2-5 years | | Every 2-3 months | | | - |
|  | Yearly | | Other, please specify: | | |  |
|  | Every 6 months | |  |  |  |  |
| **Q9.* Did you require ethical approval, informed consent or something else before**  **the start of the study?**  ***Multiple answers may be possible*** | Informed consent of each patient | | | | | *Q10* |
|  | Formal ethical approval | | | | | *If infor. cons. Is not checked: Q11* |
|  | Formal institutional approval | | | | |  |
|  | Informal institutional approval | | | | |  |
|  | Other, please specify: | | | | |  |
|  | None of the above | | | | |  |
| **Q10:* How did you obtain the informed consent?**  ***Multiple answers may be possible*** | By asking verbal agreement of each patient that came in for consultation | | | | | - |
|  | By obtaining written consent from each patient that came in for consultation | | | | |  |
|  | By putting up posters or leaflets on the study in the waiting areas | | | | |  |
|  | Other, please specify: … | | | | |  |
| **Q11: How did you collect the data?** | On paper forms, followed by data-entry in the web application | | | | | Q12 |
|  | Directly into the web-based application | | | | | Q14 |
|  | In an electronic file (e.g. Excel), followed by data-entry in the web application | | | | | Q12 |
|  | Other, please specify: … | | | | | Q12 |
| ***Section 3: Effort and Time investment*** |  | | | | |  |
| **Q12. Can you give an estimate of the total time you and your data collection team spent on data collection on the units (in hours) for the most recent survey?**  ***For example: if it was 2 persons spending 4 hours daily for 5 days it is 2 x 4 x 5 = 40). Please give your answer in hours, without any text.*** |  | | | | | - |
| **Q13. Can you estimate the average number of minutes it took you for one patient to collect the data using the paper data collection forms?** | Average number of minutes needed to collect data for one single patient **without antimicrobials** in the unit: ______________ | | | | | - |
|  | Average number of minutes needed to collect data for one single patient **with antimicrobials** in the unit: ______________ | | | | | - |
| **Q14. Can you give an estimate of the time you and your data collection team spent on online data entry (in hours) for the most recent survey?**  ***For example: if it was 2 persons spending 4 hours daily for 3 days it is 2 x 4 x 3 = 24). Please give your answer in hours, without any text.*** |  | | | | | - |
| **Q15. Can you estimate the average number of minutes it took you for one patient to enter the data online using the Global-PPS tool?** | Average number of minutes needed to enter data for one single patient **without antimicrobials** in the unit: ______________ | | | | | - |
|  | Average number of minutes needed to enter data for one single patient **with antimicrobials** in the unit: ______________ | | | | | - |
| **Q16.* How many patients did you survey?* *If you conducted the survey in more than one facility, please combine these numbers*** | <25 | | 25-50 | | | - |
|  | 51-75 | | 76-100 | | |  |
|  | 101-200 | | 201-500 | | |  |
|  | >500 | |  | | |  |
| **Q17. Among those, how many patients receiving antimicrobial prescriptions did you survey?** | <10 | 10-25 | | | 26-50 | - |
|  | 51-75 | 76-100 | | | 101-150 |  |
|  | 151-200 | >200 | | |  |  |
| **Q18.* How many healthcare professionals in your facility were involved in the Global-PPS (organization, data collection and data entry)?*** | Microbiologists: ________ | | | | | - |
|  | Infectious disease clinicians: ________ | | | | |  |
|  | Pharmacists: ________ | | | | |  |
|  | Other clinicians: ________ | | | | |  |
|  | Nurses: ________ | | | | |  |
|  | Medical students: ________ | | | | |  |
|  | Nursing students: ________ | | | | |  |
|  | Administrative staff: ________ | | | | |  |
|  | Others: ________ | | | | |  |
| ***Section 4: Data collection and data entry*** |  | | | | |  |
| **Q19.* How satisfied were you with the following Global-PPS resources?*** | Choose from: *Not at all satisfied – Not so satisfied – Somewhat satisfied – Very satisfied – Extremely satisfied – Did not use it* | | | | | - |
|  | Global-PPS protocol: ________ | | | | |  |
|  | Data collection templates: ________ | | | | |  |
|  | User (‘IT’) manual: ________ | | | | |  |
|  | Tutorial videos: ________ | | | | |  |
|  | The helpdesk (by e-mail): ________ | | | | |  |
|  | Promotion materials: ________ | | | | |  |
|  | The feedback report: _______ | | | | |  |
| **Q20. If you have answered ‘not satisfied’ or ‘somewhat satisfied’ for one or more of these resources, could you tell us how we could improve this in the future? (Please state clearly which documents/resource you are referring to)** |  | | | | | - |
| **Q21.* How satisfied were you with the online tool for Global-PPS data entry?*** | Choose from: *Not at all satisfied – Not so satisfied – Somewhat satisfied – Very satisfied – Extremely satisfied – Did not use it* | | | | | - |
| **Q22. Do you have any suggestions on how to improve the online tool for Global-PPS data entry?** | [open question] | | | | | - |
| ***Section 5: Methodology*** |  | | | | |  |
| **Q23.* Does the Global-PPS provide enough detail?**  ***In particular the content and quality of the protocol, availability of quality indicators, etc.*** | Not enough detail | | | | | - |
|  | Sufficient detail | | | | | - |
|  | Too much detail | | | | | - |
|  | Sometimes not enough detail, sometimes too much detail | | | | | - |
| **Q24. Are there any questions/answer options you would like to add, change or remove?**  ***E.g. change certain options for presenting symptoms, or biomarker options, etc.*** | [open question] | | | | | - |
| ***Section 6: Data reporting and analyses*** |  | | | | |  |
| **Q25.* Which of the following options did you use to study your facility’s PPS results?**  ***Multiple answers may be possible*** | The feedback report (in pdf) | | | | | - |
|  | The merged feedback report for multiple facilities (in pdf) | | | | |  |
|  | The data export (in Excel) | | | | |  |
|  | None of the above yet, but we will in the future | | | | |  |
|  | None of the above yet, not planning to use them | | | | |  |
| **Q26. Do you have any suggestions on how to improve the feedback reports (e.g. information that should be added, slides that are difficult to interpret, etc…)?**  **Please write up to 3 different suggestions** | 1^st^ suggestion: ________  2^nd^ suggestion: ________  3^rd^ suggestion: ________ | | | | | - |
| **Q27.* Which themes do you find most important and would you like to see back in the feedback report?**  ***Select all that apply*** | Uptake of biomarkers/POCTs/RDTs among all patients | | | | | - |
|  | Type of biomarkers/POCTs/RDTS used in patients receiving antimicrobial prescriptions | | | | |  |
|  | (Blood) Cultures taken before start of antimicrobial | | | | |  |
|  | (Certain) symptoms | | | | |  |
|  | Presumed penicillin allergy | | | | |  |
|  | Oral/IV therapy | | | | |  |
|  | Multiple antimicrobial prescriptions | | | | |  |
|  | New/ongoing/switched antimicrobial prescriptions | | | | |  |
|  | Healthcare-Associated Infections (HAI) or | | | | |  |
|  | Community-Acquired Infections (CAI) | | | | |  |
|  | Underlying morbidities | | | | |  |
|  | Defined daily doses (DDDs) | | | | |  |
|  | *For emergency/observation wards:* Admission status | | | | |  |
|  | Other, please specify: | | | | |  |
|  | None of the above | | | | |  |
| **Q28. Are there any other results you would prefer to see in the Global-PPS feedback reports?**  ***E.g. linking certain variables, such as calculating a quality indicators score*** | [Open question] | | | | |  |
| **Q29.* We are in the process of developing the interactive feedback module for the outpatient results. In this interactive feedback, you are able to choose which results you would like to see, and which filters you would apply.**  ***Please indicate the filters you would find most important in your setting.*** | Choose from the following options: *Not useful – Somewhat useful – Very useful – N/A*  Type of antimicrobial (antibiotics, antimalarials, etc.): ________  Diagnosis (pneumonia, sepsis, etc.): ________  Indication (CAI, HAI, prophylaxis): ________  Presenting symptoms (fever, diarrhea, etc.): ________  Age group (adult, child, neonate): ________  Sex (male, female, unknown): ________  Uptake of diagnostic tests (biomarker, POCT/RDT, both): ________  (Blood) Cultures taken before start of antimicrobial: ________  *In emergency/observation wards:* Admission status (admitted, suspected admission, home, etc.): ________ | | | | | - |
| **Q30.* Did the results from the Global-PPS feedback reports contribute to a better understanding of prescribing practices in your hospital?** | *Please select from a scale from 0-100 where 0 is no contribution and 100 very much contribution* | | | | | - |
| **Q31. Could you describe how the results impacted your understanding of these prescribing practices in your setting, whether positively, negatively or not at all?** | [Open question] | | | | | - |
| **Section 7 : Barriers** |  | | | | |  |
| **Q32.* What were the main challenges you encountered while conducting the PPS (multiple choice)?** | Lack of trained staff to help conduct the survey | | | | | - |
|  | Lack of time to collect all (mandatory) variables | | | | |  |
|  | Challenges in obtaining approval from the facility management | | | | |  |
|  | Issues with obtaining ethical clearance (if needed) | | | | |  |
|  | Barriers to accessing the medical records on the units (if available) | | | | |  |
|  | Difficulties to obtain patient information during consultations | | | | |  |
|  | Internet connectivity issues | | | | |  |
|  | Language barriers | | | | |  |
|  | Other challenges, please specify: | | | | |  |
|  | No major challenges | | | | |  |
| **Q33. Could you let us know how we could be of help to you to overcome these challenges?** | [Open question] | | | | | - |
| **Q34. Provide here any other suggestion or remark you have on the Global-PPS.** | [Open question] | | | | | - |

**Appendix 1:** Detailed explanation of the data collection for the outpatient Global-PPS

Collected denominator data included brief demographic data, presenting symptoms and ordered diagnostic tests (**Table 1**, **Appendix 1 & 2**), which were largely based on stakeholder input. The variable ‘ordered test’ was requested to calculate the diagnostic testing rate during the survey.

The collected numerator data, for patients receiving an antimicrobial, contained additional information on patients’ comorbidities, and several clinical practice-related questions, such as whether treatments were based on diagnostic tests, cultures taken before the antimicrobial was started, a recorded reason of prescription, a (suspected) penicillin allergy, availability of local guidelines, and guideline adherence, as adapted from the inpatient protocol and based on stakeholder input, and existing literature (**Table 1,** **Appendix 1 & 2**).^15, 16^ Guideline adherence was inquired in more detail, covering the selected drug, dosing, intended duration and route of administration, considering all aspects are important for determining adherence of the prescription.

Patient admission status was collected for all patients in emergency and observation units, to account for patients who were already admitted or awaiting admission during or shortly after the survey. The purpose of this adaptation is to avoid mixing inpatient and outpatient methodologies in emergency and observation wards, to ultimately enhance the feasibility of the PPS.

Data collection templates and the protocol are available in the Supplementary Materials. Online training sessions are held multiple times a year to provide guidance on data collection and entry.

**Appendix 2:** Data collection forms for the outpatient module of the Global-PPS

**Unit Form (Mandatory : Fill in one form for each unique unit/room included in the survey)**

| **Date of survey** (dd/mm/year) | ___/___/_____ | | **Person(s) completing form** (Auditor code) *(optional)* | |  |
| --- | --- | --- | --- | --- | --- |
| **Name institution:** |  | | **Unit Name** | |  |
|  |  |  | **Room name(s)** *(optional)* | |  |
|  |  | |  | |  |
| **Type of speciality - Tick just “one” most appropriate type of outpatient unit** | | | | | |
| - EM **(Emergency)** - OB (**Observation**) - RESP (Respiratory) - ID (Infectious disease) - HIV/TB (HIV-Tuberculosis) - REN (Nephrology-urology) - DIAL (Dialyses) - GAS (Gastroenterology) | | - GM (**General (Internal) Medicine mixed**)**^1^** - SM (**Surgical Mixed**) - HO (Haematology-Oncology) - PLAS (Plastic Reconstructive Surgical) - ORT (Orthopaedic) - ENT (Ear Nose and Throat) - EYE (Ophthalmology) - ENDO (Endoscopy) - STI (Sexually Transmitted Infection) | | - HCP (**Healthcare Practice**) - GP (**General Practitioner practise**) - MAL (Malnutrition) - NM (Neonatal Medical) - ANC (Antenatal care) - GYN (Gynaecology) - OBST (Obstetrics) - DEN (Dental Clinic) | |

| **Total number of prescribers^2^ on the unit/room during defined timeslot of the survey** | | | | | |
| --- | --- | --- | --- | --- | --- |
| **N doctor(s)** | | **N nurse(s)** | | **N pharmacist(s)** | **N other(s)** |
|  | |  | |  |  |
|  |  |  |  |  |  |
| **Timeslot data gathering on the day of the survey^4^** | | | **Starting time^3^ (hour): ­­­­­­­­**_______________  **O a.m. O p.m.** (tick as appropriate)  **Ending time^3^ (hour):** _______________  **O a.m. O p.m.** (tick as appropriate) | | |

^1^ Includes specialties such as Dermatology, Allergy-Immunology, Cardiovascular, etc. General medicine mixed refers also to paediatrics in general. Tick as well if no specialty is defined.

**^2^** Specify the profession of person(s) “prescribing” antimicrobials and the number of them included in the survey on the unit/room during the defined timeslot of the survey.

**^3^** Specify approximate starting hour (e.g. 8 a.m.) and approximate ending hour (e.g. 3 p.m.)

^4^ **Survey the unit for *at least 4 hours; or about a half a day (unless session is shorter, in which case, survey for the whole session duration)***. Preferably start the survey at the beginning of the session.

**OUTPATIENT Form: Complete for every outpatient seen on the unit/room and not admitted >24 hours or slept overnight during the timeslot of survey^1^**

| **Name/code of unit** |  | | **Name/code of the room within the unit** |  | **Unique patient identifier or sequential number** ^2^ | | | |  | | | **Survey Number** ^3^ |  |
| --- | --- | --- | --- | --- | --- | --- | --- | --- | --- | --- | --- | --- | --- |
| **Patient age group** *(tick as appropriate)* | | **O** Adult ≥18 years **O**  Child ≤17 years **O** Neonate | | | **Sex** | M, F, U | **Test ordered** *(tick as appropriate)* ^4^ | **O** Biomarker **O** POCT / RDT / malaria microscopy **O** UNK | | **Admission status** | **O** Already admitted **O** Suspected admission  **O** Referral other institution **O** Home **O** UNK | | |

| **Presenting symptoms or main reason(s) consultation on the day of the survey** *(tick if present, multiple choice, max. 6 choices)* | **O** Temperature >=38.3°C/>=101°F **O** Sub-febrile temperature (<38.3°C/<101°F) **O** Sneezing/nasal congestion/runny or stuffy nose **O** Acute cough **O** Chronic cough **O** Sore throat **O** Dyspnoea, difficult breathing **O** Ear pain  **O** Ear discharge **O** Eye discharge/red/swollen eyes **O** Chest pain  **O** Musculoskeletal pain **O** Headache **O** Fatigue/lethargy/general body weakness **O** General body pain **O** Confusion **O** Dizziness **O** Seizures **O** Diarrhea **O** Bloody diarrhea **O** Painful/frequent urination **O** Abdominal pain **O** Nausea/vomiting **O** Toothache/gum swelling **O** Limb swelling/warmth erythema  **O** Itch or other symptoms of genitals/anus **O** Skin lesions/spots **O** Wound/ulcer/burns **O** Trauma **O** Other symptom(s) **O** Unknown **O** None, other reason |
| --- | --- |

**To complete only if the outpatient was prescribed an antimicrobial during the defined time slot on the day of the survey**

| **Detailed patient age* ^5^** | | | ***Current weight**** *(in kg)* | ***Birth weight**** *(in kg, neonate only)* |  | **Penicillin allergy?** | **0** Yes, confirmed ^6^  **0** Yes, suspected  **0** No **0** UNK |  | **Cultures taken before start antimicrobial?^7^** | **0** Yes **0** No  **0** Unknown |
| --- | --- | --- | --- | --- | --- | --- | --- | --- | --- | --- |
| **Years (**≥2years) | **Months (**1-23month) | **Days** (<1month) |  |  |  |  |  |  |  |  |
|  |  |  |  |  |  |  |  |  | **If yes, which type of culture?** | **0** Blood **0** Other |

| **Treatment based on biomarker data** | **0** Yes **0** No | | | |  | **Treatment based on POCT, RDT, malaria microscropy** ^9^ | | | **0** Yes **0** No | |
| --- | --- | --- | --- | --- | --- | --- | --- | --- | --- | --- |
| **If yes, which biomarker** (CRP, PCT, WBC, serum lactate) ^8^ |  | | **Value** | **Unit** ^9^ |  | **If yes, specify which** (max. 3) ^10^ | **1** | **2** | | **3** |
|  |  |  |  |  |  |  |  |  | |  |
| **Type biological sample (**Blood/urine/other) | |  |  |  |  | **Result, specify** ^11^ | **0** Pos. **0** Neg. **0** Inc. | **0** Pos. **0** Neg. **0** Inc. | | **0** Pos. **0** Neg. **0** Inc. |

| **Underlying morbidity**  *(multiple choice, max. 3 choices)* | - None - Gastroenterological disease: inflammatory bowel disorders - Post-COVID ^12^ - Malnutrition ^13^ | - Diabetes mellitus, type 1 or 2 - Hematological or solid cancer/ Recent chemotherapy (<3months) - Trauma - Chronic hepatic disease, cirrhosis | - Immunosuppressed not oncology - Chronic lung diseases (incl. cystic fibrosis, COPD, bronchiectasis, asthma) - Chronic renal failure (incl. patients on dialysis) - Chronic cardiovascular disease | - AIDS/HIV - Patients with foreign body materials (incl. vascular and urinary catheters) - Other - Unknown |
| --- | --- | --- | --- | --- |

| **Antimicrobial (AM)** (generic) **Name** | | | **1.** | | **2.** | | **3.** | | **4.** | | **5.** | |
| --- | --- | --- | --- | --- | --- | --- | --- | --- | --- | --- | --- | --- |
| **Specify: new, ongoing, switch** ^15^ | **If ongoing/switch, where obtained?** (Here, HCF, Pharm, Self, else, U)^15^ | |  |  |  |  |  |  |  |  |  |  |
| **Single Unit Dose** ^16^ | | **Unit** (g, mg, IU, MU) ^16^ |  |  |  |  |  |  |  |  |  |  |
| **N Doses/day** ^17^ | | **Route** (O, R, I, IM, IV) ^18^ |  |  |  |  |  |  |  |  |  |  |
| **Intended duration prescription in N days/**UNK | | |  | |  | |  | |  | |  | |
| **Clinical diagnosis** (see appendix I) | | |  | |  | |  | |  | |  | |
| **Type of indication**  (see appendix II) | | |  | |  | |  | |  | |  | |
| **Reason in notes** (Yes, No, Not assessable, UNK)^19^ | | |  | |  | |  | |  | |  | |
| **Local guideline exists for diagnosis** (Y, N, NI, U) ^20^ | | |  | |  | |  | |  | |  | |
| **If yes (guideline exists), complete compliance** ^21^  **Drug according to guideline** (Y, N, NA, U) | | |  | |  | |  | |  | |  | |
| **Dosing according to guideline** (Y, N, NA, U) | | |  | |  | |  | |  | |  | |
| **Route of Adm. according to guideline** (Y, N, NA, U) | | |  | |  | |  | |  | |  | |
| **Duration according to guideline** (Y, N, NA, U) | | |  | |  | |  | |  | |  | |

**Note: *** *Detailed patient age,* *Current weight*, *Birth weight, ‘If ongoing, where previously prescribed’ are* **optional variables**.

**Explanation OUTPATIENT Form**

**^1^ Not admitted >24 hours or slept overnight during the timeslot of survey**: However, include patients on emergency and observation units awaiting transfer to an inpatient ward and may be occupy a bed >24 hours before the survey. Complete an outpatient form for these patients as well; these patients count in the numerator and denominator.

**^2^** Patient Identifier: A unique patient identifier or sequential attributed number or code which will not be included in the online database.

^3^ Survey Number: A unique non-identifiable number given by WebPPS. Leave blank but note down the number after the patient data has been recorded in the online database.

^4^ Test ordered: Specify if a biomarker, Point-of-Care Test (POCT), Rapid Diagnostic Test (RDT) or (malaria) microscopy test was ordered for this patient.

^5^  Detailed patient age: If the patient is ≥ 2 years, **specify only the number of years**, if between 1 and 23 months **specify only number of months**, if < 1 month **specify only number of days**.

^6^ Penicillin allergy confirmed: confirmed penicillin allergy is confirmed by Skin testing for penicillin allergy with penicillin G (Pen G), penicilloic acid (PA), and penicilloyl poly-L-lysine (PPL) ^^[[1]](#footnote-1)^^

^7^ Cultures taken before start antimicrobial: specify whether a culture was taken before an antimicrobial was administered to the patient. If yes, specify additionally which one: Blood culture or Other culture.

^8^ If “treatment based” on biomarker, specify which one: **CRP** (C-reactive protein), **PCT** (Procalcitonin), **WBC** (white blood cell count), or **serum lactate** (obtained from Arterial or Venous Blood Gas). Do not report a biomarker test if it did not contribute to the chosen antimicrobial treatment.

^9^ The unit for the biomarker CRP or PCT value expressed in mg/L, μg/L, ng/L, mg/dL, ng/dL, ng/mL, μg/mL, nmol/L. In thousand per microliter (μL) for WBC count (*normal number* of WBCs in the *blood* is 4,500 to 11,000 WBCs per microliter). The unit for serum lactate is expressed as mmol/L (normal range in adults: 0.5-2.2 mmol/L for venous blood; 0.5-1.6 mmol/L for arterial blood). For conversion calculator see: <http://unitslab.com/node/67> (CRP) and <http://unitslab.com/node/103> (procalcitonin); https://unitslab.com/node/152 (serum lactate)

^10^ Treatment based on POCT, RDT or malaria microscopy: Do not report any test if it did not contribute to chosen antimicrobial treatment. If Yes, specify ***up to 3 single POCT/RDT/microscopy tests:***

| - **HIV**, - **Malarial** antigen testing , - **Strep A**, - **MRSA** RDT, - **Dengue** RDT | - **TB** (includes MTB/RIF (detects MTB and rifampicin (RIF) resistance simultaneously) or MTB/XDR (detects resistance to isoniazid, fluoroquinolones, amikacin, kanamycin, capreomycin and ethionamide), - **GBS** (Intrapartum or antepartum Group B Streptococcus RDT), - **SARS-CoV-2**, **Flu/RSV** (Rapid detection and differentiation of Flu A, Flu B, or RSV), | - **HepB** (Hepatitis B), - **Scrub typhus** POCT**,** - **Syphilis** POCT, - **SH** (Sexual Health RDT), - **Other**. |
| --- | --- | --- |

^11^ Results biomarker: please indicate whether the result was **Pos.=**positive: e.g. when parasites were seen; **Neg.=**negative: e.g. when no parasites were seen; or **Incl.=**inconclusive: e.g. if it is unknown whether parasites were seen, or when insufficient high-power fields/white blood cells/RBCs were seen or counted, or when the quality control failed, or for another reason.

^12^ Post-COVID refers to symptoms lasting >2 months after initial COVID-19 infection with new symptoms developing >3 months post-infection.

^13^ Malnutrition refers to dietary deficiency which lead to lack of vitamins, minerals and other essential substances. Score illnesses as marasmus, kwashiorkor, scurvy, delayed growth, etc.

^14^ Specify by prescription/course: “**New**” refers to newly prescribed antimicrobials, not changed from a previous antimicrobial treatment that was prescribed for the same condition/complaints. “**Ongoing**” refers to antimicrobial treatments that are still continuing but are not changed by the prescriber. “**Switch**” is switched to an other antimicrobial and refers to antimicrobial treatments that were changed from a previous antimicrobial treatment that was prescribed for the same condition/complaints.

^15^  If ongoing, where prescribed: “**Here**” refers to the current attending institution. “**HCF**” (other healthcare facilities) refer to any hospital departments, outpatient clinics (including dental and day surgery clinics), or primary or community healthcare centers or general practitioner. “**Pharmacy**” refers to ‘over-the-counter’ use of antimicrobials without prescription. “**Self-medication**” refers to any previously administered antimicrobial (include all antimicrobials, exclude analgesics or painkillers, or anti-inflammatory drugs) without prescription (e.g. leftovers at home, received from family/friends).

^16^  Single Unit Dose: Numeric value for dose per administration and unit for the dose (in grams, milligrams, IU or MU)

^17^  N Doses/day^:^ If necessary provide fractions of doses: (e.g., every 16h = 1.5 doses per day, every 36h = 0.67 doses per day, every 48h = 0.5 doses per day).

^18^ Route: Routes of administration are: Oral=**O**; Rectal=**R**; Inhalation=**I**; Intramuscular=**IM**; Intravenous=**IV**.

^19^ Reason in Notes**:** A diagnosis / indication for the antimicrobial course is recorded in the patient’s documentation (treatment chart, notes, etc.).  **Y**=Yes; reason recorded in notes. **N**=No; reason not recorded in notes. **Not assessable**=Not assessable because e.g. no patient file was recorded in the institution. **UNK**=Unknown, not known whether reason was recorded in notes.

^20^ Guideline existing: A guideline can be a local, national or any other adopted guideline. **Y**=Yes; **N**=No; no guidelines for the specific indication. **NI**=No Information; because diagnosis/indication is unknown; **U**=Unknown.

^21^ Guideline compliance according to the **Drug**=type or choice of the antimicrobial; **Dosing**=the dosing of chosen antimicrobial; **Route of Adm.**=the route of administration; and **Duration**=the duration of the therapy/prophylaxes. **Y**=Yes; compliant to the guideline. **N**=No; Not compliant to the guideline. **NA**=Not Available; because information is missing in the guideline;

**Appendix I – Clinical diagnostic codes (what the clinician aims at treating)**

| **Site** | **Codes** | **Examples** |
| --- | --- | --- |
| **CNS** | **Proph CNS** | ***Prophylaxis*** for CNS (meningococcal) |
|  | **CNS** | Infections of the **C**entral **N**ervous **S**ystem |
| **EYE** | **Proph EYE** | ***Prophylaxis*** for Eye operations |
|  | **EYE** | Therapy for Eye infections e.g., Conjunctivitis, trachoma, blepharitis, keratitis |
| **ENT** | **Proph ENT** | ***Prophylaxis*** for **E**ar, **N**ose, **T**hroat including mouth (Surgical or Medical prophylaxis) |
|  | **PHAR** | Therapy for pharyngitis |
|  | **SIN** | Therapy for sinusitis |
|  | **AOM** | Acute otitis media and CSOM (Chronic Suppurative Otitis Media) |
|  | **ENT** | Therapy for **E**ar, Nose, **T**hroat infections, other than PHAR, SIN or AOM |
| **DEN** | **Proph DEN** | ***Prophylaxis*** for dental cases |
|  | **DEN** | Dental infections e.g. abscess, pulpitis, periodontal disease |
| **RESP** | **Proph RESP** | ***Prophylaxis*** for **Resp**iratory pathogens e.g. for aspergillosis |
|  | **LUNG** | Lung abscess including aspergilloma |
|  | **URTI** | **U**pper **R**espiratory **T**ract viral **I**nfections including influenza but not ENT |
|  | **Bron** | Acute **Bron**chitis or exacerbations of chronic bronchitis |
|  | **Bronch** | Acute bronchiolitis |
|  | **Pneu** | **Pneumonia** or LRTI (lower respiratory tract infections) |
|  | **COVID-19** | Coronavirus disease caused by SARS-CoV-2 infection |
|  | **TB** | Pulmonary TB – Tuberculosis / Extrapulmonary TB |
|  | **CF** | Complication of cystic fibrosis |
| **CVS** | **Proph CVS** | **C**ardiac or **V**ascular ***prophylaxis***, endocarditis prophylaxis |
|  | **CVS** | **C**ardio**V**ascular **S**ystem infections: endocarditis, endovascular device e.g pacemaker, vascular graft |
| **GI** | **Proph GI** | **G**astro-**I**ntestinal ***prophylaxis*** |
|  | **GO** | Acute Infectious Diarrhoea, gastroenteritis (ref <https://www.who.int/publications/i/item/WHO-MHP-HPS-EML-2022.02>) |
|  | **GI** | Any other Gastro-Intestinal infection |
|  | **CDIF** | *Clostridioides difficile* infection |
| **SSTBJ** | **Proph SST** | ***Prophylaxis*** for Skin and Soft Tissue, impetigo, plastic or orthopaedic surgery |
|  | **SST** | **S**kin and **S**oft Tissue: Cellulitis, impetigo, erysipelas, folliculitis, other viral exanthems, burn wound- and bite-related infections. |
|  | **Sys-DI** | Disseminated infection (viral infections such as measles, Cytomegalovirus …) |
|  | **DST** | **D**eep **S**oft **T**issue not involving bone e.g., infected pressure or diabetic ulcer, abscess |
| **UTI** | **Proph UTI** | ***Prophylaxis*** for recurrent **U**rinary **T**ract **I**nfection (Medical Prophylaxis) |
|  | **Cys** | Lower Urinary Tract Infection (UTI), cystitis |
|  | **Pye** | Upper UTI including catheter related urinary tract infection, pyelonephritis |
|  | **ASB** | Asymptomatic bacteriuria |
| **GUOB** | **Proph OBGY** | ***Prophylaxis*** for **OB**stetric or **GY**naecological surgery (MP: carriage of group B streptococcus) |
|  | **OBGY** | **Ob**stetric/**Gy**naecological infections, **S**exually **T**ransmitted **D**iseases (**STD**) in women, vaginitis, vaginosis |
|  | **GUM** | **G**enito-**U**rinary **M**ales + Prostatitis, epididymo­orchitis, STD in men |
| **No defined site (NDS)** | **BAC** | Bacteraemia or fungaemia with no clear anatomic site and no shock |
|  | **SEPSIS** | Sepsis of any origin (eg urosepsis, pulmonary sepsis etc), sepsis syndrome or septic shock with no clear anatomic site. Include fungaemia (candidemia) with septic symptoms |
|  | **Typh-fever** | Typhoid fever/enteric fever |
|  | **Malaria** |  |
|  | **HIV** | *Human immunodeficiency virus* |
|  | **PUO** | **P**yrexia of **U**nknown **O**rigin - Fever syndrome with no identified source or site of infection |
|  | **LO-LYMPH** | Localized acute lymphadenitis |
|  | **LYMPH** | **Lymph**atics as the primary source of infection. Suppurative lymphadenitis |
|  | **Other** | Antimicrobial prescribed with documentation but no defined diagnosis group |
|  | **MP-GEN** | Drug is for **M**edical ***Prophylaxis*** in **gen**eral, targeting no specific site, e.g. antifungal prophylaxis |
|  | **UNK** | Completely **Unk**nown Indication |
|  | **PROK** | Antimicrobial (e.g. erythromycin) prescribed for **Prok**inetic use |

**APPENDIX II - Type of Indication**

| **CAI** Community acquired infection | **Concerns any infection** acquired in the community, thus outside the healthcare setting in a patient without recent (<48hours) health care exposure. | | |
| --- | --- | --- | --- |
| **HAI**  Healthcare Associated  Infection following  **admission and/or intervention during** **hospital stay** | **HAI1** Post-operative surgical site infection (within: 30 days of surgery OR; 90 days after implant surgery) | | |
|  | **HAI2** The patient has been **discharged from hospital < 48 hours** and **has a known hospital infection or a new infection < 48 hours after discharge from hospital**. The infection can be an intervention related (e.g. intravenous or urinary catheter-related) or any other hospital acquired infection of mixed or undefined origin. | | |
|  | **HAI3** *C. difficile* associated diarrhoea (CDAD) (>48 h post-admission or <30 days after discharge from previous admission episode). | | |
| **SP** Surgical prophylaxis***** | **SP1** Single dose | **SP2** one day | **SP3**  >1 day |
| For **surgical patients** the duration of prophylaxis should be encoded as either prescription of one dose, one day (= multiple doses given within 24 hours) or prescribed >1 day. | | | |
| **MP** Medical prophylaxis | For example long term use to prevent UTI’s or penicillin in asplenic patients *etc*. | | |
| **OTH** Other | For example erythromycin as a motility agent (motilin agonist). | | |
| **UNK** | Completely unknown indication | | |

**Select 1 possibility for each reported antimicrobial**

*Surgical prophylaxis includes those antibiotics prescribed on the day of the survey for a **day-case surgical intervention, including dental procedures**.

**Appendix III: Combination anti-infective agents**

Combinations of an antibiotic and a beta-lactamase inhibitor:

Ampicillin and beta-lactamase inhibitor: report only ampicillin dose (J01CR01)

Amoxicillin and beta-lactamase inhibitor: report only amoxicillin dose (J01CR02)

*Example:*

Amoxicillin and beta-lactamase inhibitor 1.2g IV 🡪 1g (amoxicillin) + 200mg (clavulanic acid), **report 1 g as a dose**

Other combinations of multiple antimicrobial substances:

J01EE01 Sulfamethoxazole and Trimethoprim: **report the total amount of sulfamethoxazole and trimethoprim**

Example: Co-trimoxazole 960mg: (sulfamethoxazole. 800mg + trimethoprim 160mg), **report 960mg**

**Appendix 3:** Protocol for the outpatient module of the Global-PPS

# 1. General information about this protocol

The protocol sometimes refers to the data collection forms and appendices. These documents are all available as separate documents to this protocol (see [**§1.1 Outpatient forms**](file:///N:\FGEN\VAXINFECTIO\Projecten%20Prof%20H.%20Goossens\ESAC\BIOMERIEUX-PPS\protocol-development\2024\Outpatient\Protocol\Protocol%20Global-PPS%20outpatient%20module_Aug2024_clean-layoutupdate2.docx#_Outpatient_forms)).

We update our protocol incidentally to adapt to the feedback we receive. Please find all changes since the release of the outpatient module in May, 2023, under [**§1.2 Changes in the protocol versions**](file:///N:\FGEN\VAXINFECTIO\Projecten%20Prof%20H.%20Goossens\ESAC\BIOMERIEUX-PPS\protocol-development\2024\Outpatient\Protocol\Protocol%20Global-PPS%20outpatient%20module_Aug2024_clean-layoutupdate2.docx#_1.2_Changes_in).

## **1.1 Outpatient forms**

The data collection forms and appendices are available as separate documents to this protocol. In total, there are three different forms

- The **unit form *print 1x for each unit***
- The **outpatient form *print 1x for each patient***
- **Short outpatient form** for patients ***not on antimicrobials print 1x for every 4 patients***

**Please note:** You can use the **outpatient form** for patients on antimicrobials and patients not on antimicrobials. For the latter, you do not have to fill in the entire form. Therefore, you can also opt for the **short outpatient form**, where you can fill in 4 patients (not on antimicrobials) on 1 single form.

All forms are available for download under <https://www.global-pps.com/documents/>

- The **unit form** and the **outpatient form** are available in the same document: ***Data collection forms Global-PPS outpatient module***
- **Short outpatient form** for patients ***not on antimicrobials*** is available in a separate document: ***Short version G-PPS data collection forms for outpatients not on antimicrobials***

## **1.2 Changes in the protocol versions**

**November 2023 version of protocol:**

- Clarification of exclusion criteria

**May 2024 version of protocol:**

- Addition of new variables measuring the quality of prescription:

(1) Test ordered

(2) Penicillin allergy

(3) If prescription was ongoing/switched, where was it obtained

(4) Reason documented in notes

(5) Route of administration according to guidelines

- Adaptation of existing variables:

For the variable *Presenting symptoms*, some additional symptoms have been added and others adapted

Serum lactate is added as an option to *Treatment based on biomarker*

Malaria microscopy is added as an option to *Treatment based on POCT/RDT*

Up to 3 POCT/RDTs can now be selected

For the variable *Underlying morbidity*, some additional morbidities have been added and others adapted.

**August 2024 version of protocol:**

- Addition of new variable measuring the quality of prescriptions:

1. Cultures taken before start antimicrobial

# 2. Background and aims

The **Global Point Prevalence Survey** (Global-PPS or G-PPS) provides a simple, freely available web-based tool to measure and monitor antimicrobial prescribing in institutions worldwide. The Global-PPS has established a global network of institutions conducting point prevalence surveys and provides quantifiable measures to assess and compare quantity and quality of antimicrobial prescribing in inpatient and outpatient adults, children and neonates worldwide.

The Global-PPS was first piloted in 2014, with worldwide studies conducted in 2015^[[2]](#footnote-2)^ and 2017. Since 2018, three survey periods a year are available. The Global-PPS is coordinated at the University of Antwerp, Belgium and sponsored through an unrestricted grant given to them annually by bioMérieux.

This outpatient Global-PPS protocol is produced thanks to the cooperation of [several Global-PPS participants](https://www.global-pps.com/acknowledgements/) . Their valuable comments and recommendations were taken into account when drafting this protocol. For the first time, it is also possible to monitor antimicrobial prescribing among patients in outpatient care facilities using the method of a point prevalence survey. This protocol is an extension of the existing Global-PPS protocol to monitor inpatients.

The outpatient module offers a trustworthy way to collect antimicrobial use data in outpatients in high- as well as low- and middle- income countries. Participating institutions receive extensive, high-quality information about prescribing patterns in their outpatient units.

Please find below our aims ([**§2.1 Main aims**](file:///N:\FGEN\VAXINFECTIO\Projecten%20Prof%20H.%20Goossens\ESAC\BIOMERIEUX-PPS\protocol-development\2024\Outpatient\Protocol\Protocol%20Global-PPS%20outpatient%20module_Aug2024_clean-layoutupdate2.docx#_2.1_Main_aims)) and core benefits to participants ([**§2.2 Core benefits**](file:///N:\FGEN\VAXINFECTIO\Projecten%20Prof%20H.%20Goossens\ESAC\BIOMERIEUX-PPS\protocol-development\2024\Outpatient\Protocol\Protocol%20Global-PPS%20outpatient%20module_Aug2024_clean-layoutupdate2.docx#_2.2_Core_benefits)).

## **2.1 Main aims**

The main aims of the outpatient Global-PPS, is to aid and support participants in the following:

- Surveillance of performance indicators and ***identify targets for quality improvement*** ***of antimicrobial prescribing*** *→* ***identify burden***
- Designing tailor-made interventions to ***promote prudent use of antimicrobials*** *→* ***change practice***
- Assessing the ***effectiveness of such interventions***, through repeated PPS *→* ***measure impact***

The Global-PPS tool supports the concept of simplicity and feasibility by providing a user-friendly hands-on tool that can be repeated easily to support stewardship programs.

## **2.2 Core benefits**

- The web-based tool supports **real-time data collection**, is **easy to use**, requires minimal training, speeding up and simplifying data entry;
- The institution (hospital or healthcare facility**)** will be able to download a **real-time one-point report** which can be used for local communications and presentations (since 2024);
- There is evidence of consistency and reproducibility with the data entry using this tool;
- Participation in the survey has **encouraged thorough engagement and feedback**, enhancing communication between prescribers and the local infectious diseases colleagues;
- The Global-PPS **enables sharing of best practices** and **raises awareness of inappropriate antimicrobial prescribing** with broad adaptability and suitability for a range of healthcare resource settings.

# 3. Methods and protocol specifics

The Global-PPS outpatient module is a cross-sectional survey, collecting outpatient antimicrobial prescribing information in a short period. For more details, please read below our [**§3.1 Inclusion and exclusion criteria**](file:///N:\FGEN\VAXINFECTIO\Projecten%20Prof%20H.%20Goossens\ESAC\BIOMERIEUX-PPS\protocol-development\2024\Outpatient\Protocol\Protocol%20Global-PPS%20outpatient%20module_Aug2024_clean-layoutupdate2.docx#_3.1_Inclusion_and), [**§3.2 Timeframe of surveillance**](file:///N:\FGEN\VAXINFECTIO\Projecten%20Prof%20H.%20Goossens\ESAC\BIOMERIEUX-PPS\protocol-development\2024\Outpatient\Protocol\Protocol%20Global-PPS%20outpatient%20module_Aug2024_clean-layoutupdate2.docx#_2.1_How_to) and [**§3.3 Denominator and numerator data**](file:///N:\FGEN\VAXINFECTIO\Projecten%20Prof%20H.%20Goossens\ESAC\BIOMERIEUX-PPS\protocol-development\2024\Outpatient\Protocol\Protocol%20Global-PPS%20outpatient%20module_Aug2024_clean-layoutupdate2.docx#_3.3_Denominators_and).

## **3.1 Inclusion and exclusion criteria**

In the Global-PPS outpatient module, it is very important to survey units where outpatients are seen. More detailed inclusion criteria for the unit ([**§3.1.1 Unit-level criteria**](file:///N:\FGEN\VAXINFECTIO\Projecten%20Prof%20H.%20Goossens\ESAC\BIOMERIEUX-PPS\protocol-development\2024\Outpatient\Protocol\Protocol%20Global-PPS%20outpatient%20module_Aug2024_clean-layoutupdate2.docx#_3.1.1_Unit-level_criteria)), patient ([**§3.1.2 Patient-level criteria**](file:///N:\FGEN\VAXINFECTIO\Projecten%20Prof%20H.%20Goossens\ESAC\BIOMERIEUX-PPS\protocol-development\2024\Outpatient\Protocol\Protocol%20Global-PPS%20outpatient%20module_Aug2024_clean-layoutupdate2.docx#_3.1.2_Patient-level_criteria)) and antimicrobial prescription ([**§3.1.3 Antimicrobial-level criteria**](file:///N:\FGEN\VAXINFECTIO\Projecten%20Prof%20H.%20Goossens\ESAC\BIOMERIEUX-PPS\protocol-development\2024\Outpatient\Protocol\Protocol%20Global-PPS%20outpatient%20module_Aug2024_clean-layoutupdate2.docx#_3.1.3_Antimicrobial-level_criteria)) can be found below.

### 3.1.1 Unit-level criteria

The following units/departments and institutions (hospital or healthcare facility) can be monitored using the Global-PPS outpatient module:

- **Emergency**, **outpatient departments** and **day surgery departments** of hospitals
- **Outpatient clinics** or **out-of-hospital clinics** such as primary care clinics, urgent care clinics, ambulatory surgery centres including day surgery units not requiring overnight admission or stay.
- **Primary healthcare centres** or **community health centres for outpatients** that:
  - have a limited number of inpatients beds available, and/or
  - have observation beds for patients not staying overnight, or
  - have no inpatients or observation beds available.

**Important**: **All outpatient units or rooms** within the participating facility should preferably included if you participate for the very first time in the outpatient Global-PPS, in order to create a baseline for your antimicrobial prescribing patterns.

Please survey the units **for at least 4 hours** (see also: Timeframe). You can survey different units on different days.

### 3.1.2 Patient-level criteria

Include **all outpatients** seen in your survey. In the Global-PPS, we define outpatients as:

´Patients who visit a healthcare facility for diagnosis or treatment but ***do not stay overnight***. Their visits include regular check-ups, minor surgeries, routine tests and certain therapies like dialysis.’

**Important exception**: Patients on specific units such as **emergency units** (wards) or **observation units** (wards) should also be included if they are still present on the unit and occupying a bed during the timeframe of surveillance on the day of the PPS.

For these patients, an outpatient form should be completed as well, with an additional variable: *admission status*. These patients should be included because it can be difficult to distinguish ‘true’ inpatients and outpatients in these wards.

**Important**: **Selecting patients** (based on clinical signs, symptoms, or diagnosis) **is not allowed!**

### 3.1.3 Antimicrobial-level criteria

Include all **newly** **prescribed** (during the survey), **ongoing** and **switched antimicrobial prescriptions**. An ***ongoing or switched antimicrobial*** is an antimicrobial that was prescribed prior to the study, and ***was not stopped during the survey***. This includes antimicrobials that are taken e.g. every 48 hours (even if the patient did not take the antimicrobial on the day of the survey).

Include all of the following antimicrobial types (according to the WHO ATC classification^[[3]](#footnote-3)^):

- ***Antibacterials for systemic use***: J01
- ***Antimycotics and antifungals for systemic use***: J02 & D01BA (including griseofulvin and terbinafine)
- ***Drugs for treatment of tuberculosis***: J04A (these are the antibiotics as well as all other drugs to treat tuberculosis)
- ***Antibiotics used as intestinal anti-infectives***: A07AA
- ***Antiprotozoals used as antibacterial agents****,* ***nitroimidazole derivatives:*** P01AB
- ***Antivirals for systemic use***: J05
- ***Antimalarials***: P01B

The list of all included antimicrobials is available at [www.global-pps.com/documents](http://www.global-pps.com/documents/), which contains all substances with their route of administration. If an antimicrobial is not present in this list, please contact [global-pps@uantwerpen.be](mailto:Global-PPS@uantwerpen.be).

**Important**: **Exclude** antimicrobials for **topical use!**

## **3.2 Timeframe of surveillance**

For the timing of the survey, please consider both the period, i.e. the month or season (see [**§3.2.1 Period of surveillance**](file:///N:\FGEN\VAXINFECTIO\Projecten%20Prof%20H.%20Goossens\ESAC\BIOMERIEUX-PPS\protocol-development\2024\Outpatient\Protocol\Protocol%20Global-PPS%20outpatient%20module_Aug2024_clean-layoutupdate2.docx#_3.2.1_Period_of)), and timeslot, i.e. the day of the week and hour of the day (see [**§3.2.2 Timeslot of surveillance**](file:///N:\FGEN\VAXINFECTIO\Projecten%20Prof%20H.%20Goossens\ESAC\BIOMERIEUX-PPS\protocol-development\2024\Outpatient\Protocol\Protocol%20Global-PPS%20outpatient%20module_Aug2024_clean-layoutupdate2.docx#_3.2.2_Timeslot_of)), as both factors can influence the antimicrobial prescribing patterns that you measure.

### 3.2.1 Period of surveillance

Data should be collected within three predefined timeframes a year:

- **January-April**
- **May-August**
- **September-December**

Data collection should be finished within the period it was started. Preferably, complete the data collection within a few weeks from the start, to minimize differences in antimicrobial prescribing patterns related to seasonal changes.

**Important**: Units can be surveyed multiple times within a survey (which differs with the inpatient module)

### 3.2.2 Timeslot of surveillance

Survey your unit or room on a **day that represents your usual practice**, such as a regular week day. If you choose a holiday or a day in the weekend, you might measure antimicrobial prescribing practices that differ from your usual practice, and therefore could not be generalized for a longer period.

Survey your unit **for at least 4 hours**, to capture a sufficient number of patients with antimicrobial prescriptions that are representative of your setting. Sometimes, you might need to survey for a longer period of time, for example a full day or a couple of days, to capture this sufficient number of patients.

**Sufficient number of patients**: It is important that you capture a ‘sufficient number of patients’ to represent your usual setting. We do not specify a minimum number of patients to collect, this **depends on what is feasible in your setting and the aim of your survey**.

For example, for an initial survey to familiarize yourself with the method, you can capture less patients than when you want an in-depth analyses to develop targets to improve the quality of antimicrobial prescribing in your facility.

Please keep in mind that the more patients (with antimicrobial prescriptions) you capture, the more representative this sample is for your setting.

## **3.3 Denominators and numerator data**

In the Global-PPS, antimicrobial prevalences are calculated using numerator and denominator data. For the outpatient Global-PPS, these data are collected at patient-level (which differs compared with the inpatient module):

- **The denominator** = the total number of patients seen in the survey.

This is collected by asking general information for each patient seen in the survey (see [**§User manuals & Tutorial videos**](file:///N:\FGEN\VAXINFECTIO\Projecten%20Prof%20H.%20Goossens\ESAC\BIOMERIEUX-PPS\protocol-development\2024\Outpatient\Protocol\Protocol%20Global-PPS%20outpatient%20module_Aug2024_clean-layoutupdate2.docx#_4.1_User_manuals)).

- **The numerator** = the total number of patients receiving an antimicrobial prescription in the survey

This is collected by asking more detailed information for each patient on antimicrobials in the survey (see [**§User manuals & Tutorial videos**](file:///N:\FGEN\VAXINFECTIO\Projecten%20Prof%20H.%20Goossens\ESAC\BIOMERIEUX-PPS\protocol-development\2024\Outpatient\Protocol\Protocol%20Global-PPS%20outpatient%20module_Aug2024_clean-layoutupdate2.docx#_4.1_User_manuals)).

# 4. Data collection forms

Two methods of data collection are possible:

- **Data collection on paper forms, before entry in the online tool**

Please print all forms as described in [**§1.1 Outpatient forms**](file:///N:\FGEN\VAXINFECTIO\Projecten%20Prof%20H.%20Goossens\ESAC\BIOMERIEUX-PPS\protocol-development\2024\Outpatient\Protocol\Protocol%20Global-PPS%20outpatient%20module_Aug2024_clean-layoutupdate2.docx#_1.1_Outpatient_forms).

- **Direct data entry in the online tool**

Please make sure all data are readily available, because you need to complete the full unit and patient forms before being able to save them. Find more information in [**§5. Data entry**](file:///N:\FGEN\VAXINFECTIO\Projecten%20Prof%20H.%20Goossens\ESAC\BIOMERIEUX-PPS\protocol-development\2024\Outpatient\Protocol\Protocol%20Global-PPS%20outpatient%20module_Aug2024_clean-layoutupdate2.docx#_5._Data_entry) and [**§User manuals & Tutorial videos**](file:///N:\FGEN\VAXINFECTIO\Projecten%20Prof%20H.%20Goossens\ESAC\BIOMERIEUX-PPS\protocol-development\2024\Outpatient\Protocol\Protocol%20Global-PPS%20outpatient%20module_Aug2024_clean-layoutupdate2.docx#_4.1_User_manuals).

## **4.1 The Unit form**

The Unit form contains all the following variables. All variables marked with * are mandatory:

- **Date of survey***: The data on which the unit, or room belonging to the unit, is surveyed: dd/mm/yy
- **Auditor code**: The code, initials or else, of the person completing the form. This can be used to help adding or correcting entered data, or to track possible bias linked to the auditor.
- **Institution name***: Name of the institution (hospital or healthcare facility)
- **Unit name***: Unique name of the unit/department/ward.
- **Room name**: Unique name of the room belonging to a particular unit. Of note, a unit can have several rooms. Define these uniquely!
- **Unit Type of specialty***: Defines the most appropriate type of outpatient unit. Select only one type. The complete list is available in the outpatient data collection forms, p. 1, and in this protocol p. 9.
- **Total number of prescribers on the unit/room during defined timeslot of the survey***: Count the number of doctors, nurses, pharmacists and/or others who were prescribing the antimicrobials to the outpatients during the timeslot of the survey. Fill in the number 0 if (one of these) prescribers are not present during the timeslot of the survey. Fill in 0 if the healthcare professionals were present, but not prescribing antimicrobials.
- **Timeslot of data gathering on the day of the survey*****:** Specify the approximate starting time and ending time (in hours and minutes AM or PM) on the day of the survey. The unit must be ***surveyed for at least 4 hours or about half a day***, unless the duration of the consultation session is shorter, then survey for the whole session duration. Preferably start the survey at the beginning of the session.

## **4.2 The Patient form**

The Patient form contains three different sections:

| 1. [**§4.2.1 General patient information**](file:///N:\FGEN\VAXINFECTIO\Projecten%20Prof%20H.%20Goossens\ESAC\BIOMERIEUX-PPS\protocol-development\2024\Outpatient\Protocol\Protocol%20Global-PPS%20outpatient%20module_Aug2024_clean-layoutupdate2.docx#_4.2.1_General_patient) | for ***all patients seen during the timeslot*** of survey (=denominator) |
| --- | --- |
| 1. **[§4.2.2 Detailed patient information](file:///N:\\FGEN\\VAXINFECTIO\\Projecten%20Prof%20H.%20Goossens\\ESAC\\BIOMERIEUX-PPS\\protocol-development\\2024\\Outpatient\\Protocol\\Protocol%20Global-PPS%20outpatient%20module_Aug2024_clean-layoutupdate2.docx#_4.2.2_Detailed_patient)** | ***for each patient on antimicrobials*** (=numerator) |
| 1. [**§4.2.3 Antimicrobial treatment information**](file:///N:\FGEN\VAXINFECTIO\Projecten%20Prof%20H.%20Goossens\ESAC\BIOMERIEUX-PPS\protocol-development\2024\Outpatient\Protocol\Protocol%20Global-PPS%20outpatient%20module_Aug2024_clean-layoutupdate2.docx#_4.2.3_Antimicrobial_treatment) |  |

### 4.2.1 General patient information

The ‘General patient information’ part of the Patient form contains all the following variables. All variables marked with * are mandatory:

- **Unit (Name/code)***: This is the unique **name** of the unit studied. This name is selected using the drop-down list in the Global-PPS tool, as this outpatient unit name has been defined during the preparation of the department list at institutional level (see [**§5.2 Prepare the department list**](file:///N:\FGEN\VAXINFECTIO\Projecten%20Prof%20H.%20Goossens\ESAC\BIOMERIEUX-PPS\protocol-development\2024\Outpatient\Protocol\Protocol%20Global-PPS%20outpatient%20module_Aug2024_clean-layoutupdate2.docx#_5.2_Prepare_the))
- **Room (Name/code):** This is the unique name of a room within the unit. If the unit does not contain separate rooms, please use the same room name for all patients or leave the room name blank. *Optional field.*
- **Unique Patient Identifier***: This is a unique number allowing local tracing at patient-level for any clarifications (e.g. the clinical record/note number, sequential number). This information will not (and cannot) be reported or submitted in the Global-PPS database.
- **Survey Number***: It’s a unique non-identifiable number generated by the Global-PPS tool for each patient record. Please ensure that the person entering the data online ***writes down this number immediately when it is generated by the tool as it will not be displayed again***. This number identifies the patient uniquely in the Global-PPS database, and starts with OP-[number]. Important, this number will be generated when all data has been correctly entered online and will be given to you after clicking:


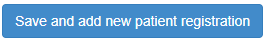


Only then, the patient is stored in the database !

- **Patient age group***: This is the age category of the patient. Choose one of three options: **adults** (≥18 years old), **child** (≤17 years old) or **neonate** (≤30 days old).
- **Sex***: This is the sex of the patient. Choose one of three options: **Male**, **Female** or **Unknown**.
- **Test ordered***: This is whether any biomarker or Point-of-Care test/Rapid Diagnostic Test/malaria microscopy test was ordered for this patient for the ***same presenting complaints***. This is independent of whether the results were available yet and whether the treatment was based on these tests. The test can be ordered on the same day as the visit, or a few days before if the patient had visited this institution for the same complaints before, as long as those tests were ordered for the same complaints.
- **Admission status***: For **emergency and observation units**, it is requested to also register patients who slept overnight due to specific reasons, e.g. they are waiting for transfer to another institution or ward and are still present during the timeslot of survey (see [**§3.1.2 Patient-level criteria**](file:///N:\FGEN\VAXINFECTIO\Projecten%20Prof%20H.%20Goossens\ESAC\BIOMERIEUX-PPS\protocol-development\2024\Outpatient\Protocol\Protocol%20Global-PPS%20outpatient%20module_Aug2024_clean-layoutupdate2.docx#_3.1.2_Patient-level_criteria)). For these specific units only, the ***status of admission needs to be recorded “as decided during the 4-hour survey period (see timeslot)”***. There are 5 possibilities:
- **already admitted** = decision of admission is taken
- **suspected admission** = awaiting for admission, final decision not yet taken
- **referral other institution**
- **home**
- **Unknown** (UNK)
- **Presenting symptoms or reason of consultation on the day of the survey*:** These are the presenting symptoms of a patient on the day of the survey. Choose at least 1 and maximum 6 symptoms (see outpatient data collection form, page 2).

### 4.2.2 Detailed patient information

The ‘Detailed patient information’ part of the Patient form must be completed **only if the patient had a new/ongoing/switched antimicrobial prescription during the survey**. This form contains all the following variables. All variables marked with * are mandatory:

- **Age**: Three fields, one for the year, one for the month and one for the days, are available. ***Only one of these fields needs to be completed as follows:***
  - If ≤ 30 days old, write the exact numbers of days.
  - For patients older than 1 month and younger than 2 years, fill in month field. (e.g. 19 months)
  - If the patient is at least 2 years old, then only the year field is to be recorded.
- **Current weight**: Write the current weight in Kg with one decimal number (*Optional field)*
- **Birth weight**: Write the birth weight in Kg with one decimal number.  **Only for neonates** *(optional field)*
- **Treatment based on biomarker data or white blood cell count (WBC)*:** Tick ‘**Yes**’ or ‘**No**’. It refers to whether or not biomarker results are used to initiate the antibiotic treatment. If yes, next lines should also be completed with 4 possible answers (report the most relevant one):
- **CRP** = in case the treatment is based on results of CRP (*C-reactive protein)*
- **PCT** = in case the treatment is based on results of PCT (*procalcitonin*)
- **WBC** = in case the treatment is based on elevated white blood cell count. Normal number of WBCs in the blood is ± 4,500 to 11,000 WBCs per microliter.
- **Serum lactate** = in case the treatment is based on Arterial or Venous Blood Gas lactate. Normal range in adults: 0.5-2.2 mmol/L for venous blood; 0.5-1.6 mmol/L for arterial blood.
- **Type of biological fluid sample*:** choose between Blood, Urine or Other.

Complete if available also the ***most relevant value close to the start of the antibiotic treatment*** (numeric optional field) in **mg/L**, **μg/L**, **ng/L**, **mg/dL**, **ng/dL**, **ng/mL**, **μg/mL**, **nmol/L**. In thousand per microliter (μL) for WBC count.

For conversion calculator see: <http://unitslab.com/node/67> (CRP) and <http://unitslab.com/node/103> (procalcitonin).

- **Treatment based on POCT (Point of Care Test) or RDT (Rapid Diagnostic Test) or malaria microscopy***: Tick ‘**Yes**’ or ‘**No**’. It refers to whether or not POCT/RDT/microscopy results are used to initiate the antibiotic treatment. If yes, specify up to 3 POCTs or RDTs (report the most appropriate one) (see list outpatient data collection form, page 3).
- **Cultures taken before start of antimicrobial***: Tick ‘**Yes**’, ‘**No**’ or ‘**Unknown**’. It refers to whether a was taken before an antimicrobial was administered to the patient. If yes, specify which type of culture was taken (blood, other).
- **Underlying morbidity***: Refers to underlying morbidities a patient has at time of the survey. Select at least 1 and maximum 3 choices (see list outpatient data collection form, page 2).

### 4.2.3 Antimicrobial treatment information

The ‘Antimicrobial treatment information’ part of the Patient form must be completed **only if the patient had a new/ongoing/switched antimicrobial prescription during the survey**. This form contains all the following variables. All variables marked with * are mandatory:

- **Antimicrobial Drug Name**: This is the generic name (e.g. amoxicillin and beta-lactamase inhibitor and not Augmentin®). Antimicrobials for topical use applied on the skin/eye/ear etc. are not included. The antimicrobial data are automatically recorded in the Global-PPS tool with its ATC5 code following the ATC classification system of the WHO Collaborating Centre for Drug Statistics. (<https://www.whocc.no/atc_ddd_index/>). See **[§3.1.3 Antimicrobial-level criteria](file:///N:\\FGEN\\VAXINFECTIO\\Projecten%20Prof%20H.%20Goossens\\ESAC\\BIOMERIEUX-PPS\\protocol-development\\2024\\Outpatient\\Protocol\\Protocol%20Global-PPS%20outpatient%20module_Aug2024_clean-layoutupdate2.docx#_3.1.3_Antimicrobial-level_criteria)** for antimicrobials to include.
- **Prescription/course**: Tick ‘**New**’ or ‘**Ongoing’** or ‘**Switch’**. It refers to whether the antimicrobial treatment is first prescribed on the day of the survey (‘new’), or if it was already prescribed before and not stopped during the day of the survey (‘ongoing’), or whether it was switched from an already existing antimicrobial (‘switch’).
- **If ongoing/switch**, **where was the prescription obtained?**: This refers to where the original prescription was obtained for patients who visited your institution with already an antimicrobial prescription. Tick ‘**Here**’, ‘**Other healthcare facility’**, ‘**Pharmacy**’, ‘**Self-administered**’, ‘**Else**’ or ‘**Unknown**’.
- “**Single Unit Dose**” **and** “**Unit**” **of Dose**: Administered dose is the actual prescribed single unit dose per administration, expressed in mg, g, IU or MU. Provide number of times/day given in next variable (doses/day)

**Combinations with enzyme inhibitors**: For combination with **one active ingredient** as the main antimicrobial agent, like penicillins with beta-lactamase inhibitors, only the **content of active ingredient** should be recorded and entered in the Global-PPS tool. E.g. amoxicillin and beta-lactamase inhibitor 500/125 (amoxicillin 500 mg and clavulanic acid 125 mg as potassium salt) should be entered as 500 mg. Important: this must still be recorded as amoxicillin and beta-lactamase inhibitor and NOT amoxicillin!

**Other examples: (**<https://atcddd.fhi.no/ddd/list_of_ddds_combined_products/>**)**

- J01CR01 Ampicillin and beta-lactamase inhibitor: report only ampicillin dose
- J01CR02 Amoxicillin and beta-lactamase inhibitor: report only amoxicillin dose
- J01CR03 Ticarcillin and beta-lactamase inhibitor: report only ticarcillin dose
- J01CR05 Piperacillin and beta-lactamase inhibitor: report only piperacillin dose

**Combinations of two active ingredients**: For combination of **two or more active ingredients** as antimicrobial agents, like sulfamethoxazole and trimethoprim, the t**otal content should be entered** in Global-PPS tool. For example, sulfamethoxazole 200 mg/trimethoprim 40 mg will be recorded as 240 mg.

- **Doses per Day**: This refers to the number of actual prescribed doses per 24 hours. This can be calculated by: ***N doses / N days***, e.g. 1 dose per 2 days = 0.5 dose per day. For example every 6 hours = 4 doses/day, every 8h = 3 doses/day, every 12h = 2 doses/day, every 16h = 1.5 doses/day, every 36h = 0.67 doses/day, every 48h = 0.5 doses/day, and every 72h = 0.33 doses/day.
- **Route**: Route of Administration. Five routes of administration are included: **Oral=O**, **Intravenous and intrathecal and intraperitoneal=P**, **Intramuscular=IM**, **Rectal=R**, **Inhalation=I**. For analyses intravenous, intrathecal and intramuscular are all parenteral use (=P).
- **Prescribed / intended duration in N days or UNK**: This refers to number of days the antimicrobial is prescribed. Specify the number of days (if it exceeds 100 days, please write down 100 days).
- **Clinical diagnosis**: This is the reason to treat the patient (see appendix I, page 4 of outpatient data collection forms). Select ***ONLY ONE*** of the possibilities. If more categories are possible, write the one most applicable. Request additional information from doctors, nurses or pharmacists if needed.
- **Type of indication**: Refers to whether it concerns therapeutic treatment (**Community Acquired Infection=CAI or Healthcare-Associated Infection=HAI**) or prophylactic use (**Medical or Surgical prophylaxis**). The indication should be obtained from ward staff if missing (See appendix II, page 5 of outpatient data collection forms for available codes).

**Intended duration for surgical prophylaxis**: The **intended duration** of antibiotics for surgical prophylaxis can be filled in as (1) **one-dose (SP1)**, (2) **one day (=multiple doses given in one day, SP2)**, or (3) **>1 day** **(SP3)**.

- **Reason in notes**: Refers to whether ***the reason for the antimicrobial treatment was recorded*** in the (notes of the) patient file. Tick ‘**Yes**’, ‘**No**’, ’**Not** **assessable**’.
- **Local guidelines exist**: This refers to (e.g. local/national/WHO) ***guidelines used in the institution***. Tick **Y=Yes**; **N=No** guidelines for the specific indication; **NI=No Information** because diagnosis/indication is unknown; **U=Unknown**.
- **Guideline compliance**: depending on whether a local guideline exist, four additional variables can be completed, about guideline compliance according to :
  - the ***type or choice of the antimicrobial***
  - the ***dosing***
  - the ***duration of the therapy/prophylaxis***
  - the ***route of administration***

Tick : **Y=Yes**, compliant to the guideline; **N=Not compliant** to the guideline; **NI=Not indicated** because the choice of drug is not compliant according to guideline; **U=Unknown**.

# 5. Data entry

All data need to be entered in the online tool: <https://app.globalpps.uantwerpen.be/globalpps_webpps/>

Before you start entering your data, some preparatory works needs to be completed, including registration of your account (and additional users) and registration of the institution, all described below in [**§5.1 Register, login and create your institution**](file:///N:\FGEN\VAXINFECTIO\Projecten%20Prof%20H.%20Goossens\ESAC\BIOMERIEUX-PPS\protocol-development\2024\Outpatient\Protocol\Protocol%20Global-PPS%20outpatient%20module_Aug2024_clean-layoutupdate2.docx#_5.1_Register,_login).

Furthermore, all departments need to be entered (see [**§5.2 Prepare the department list**](file:///N:\FGEN\VAXINFECTIO\Projecten%20Prof%20H.%20Goossens\ESAC\BIOMERIEUX-PPS\protocol-development\2024\Outpatient\Protocol\Protocol%20Global-PPS%20outpatient%20module_Aug2024_clean-layoutupdate2.docx#_5.2_Prepare_the)) and the appropriate survey period must be selected (see [**§5.3 Select your survey period**](file:///N:\FGEN\VAXINFECTIO\Projecten%20Prof%20H.%20Goossens\ESAC\BIOMERIEUX-PPS\protocol-development\2024\Outpatient\Protocol\Protocol%20Global-PPS%20outpatient%20module_Aug2024_clean-layoutupdate2.docx#_5.3_Select_your)) before you can start entering unit and patient data (see [**§5.4 Enter unit and patient data**](file:///N:\FGEN\VAXINFECTIO\Projecten%20Prof%20H.%20Goossens\ESAC\BIOMERIEUX-PPS\protocol-development\2024\Outpatient\Protocol\Protocol%20Global-PPS%20outpatient%20module_Aug2024_clean-layoutupdate2.docx#_5.4_Enter_unit)).

**More information needed?**: Detailed instructions on how to add extra users, what to do if you lost your login, how to change your password, or how to enter departments, select your survey and enter unit and patient data, can all be found in the user manuals (see **[§User manuals & Tutorial videos](file:///N:\\FGEN\\VAXINFECTIO\\Projecten%20Prof%20H.%20Goossens\\ESAC\\BIOMERIEUX-PPS\\protocol-development\\2024\\Outpatient\\Protocol\\Protocol%20Global-PPS%20outpatient%20module_Aug2024_clean-layoutupdate2.docx#_User_manuals_&)**)

## **5.1 Register, login and create your institution**

### 5.1.1 Participate for the first time?

If your institution participates for the first time, you need to register and create your institution in the online tool. Once you register your account, make sure you confirm your registration by following the instructions in the confirmation mail.

**Important**: The person who creates the institution in the Global-PPS tool, automatically becomes the **local administrator**. Please make sure the correct person creates the institution. Made a mistake? Please contact [Global-PPS@uantwerpen.be](mailto:Global-PPS@uantwerpen.be)

All hospitals, outpatient clinics and primary healthcare centers or other healthcare facilities seeing outpatients can participate. When **registering your institution**, you need to select an ***institution type***:

- **Primary hospital**: often referred to as a district hospital or first-level referral. The hospital has ***few specialities***, mainly internal medicine, obstetrics-gynaecology, paediatrics, and general surgery, or only general practice; limited laboratory services are available for general, but not for specialized pathological analysis. Often corresponds to general hospital without teaching function.
- **Secondary hospital**: often referred to as provincial hospital. A hospital ***highly differentiated by function with five to ten clinical specialities*** including some haematology, oncology, renal and ICU beds; takes some referrals from other (Primary) hospitals. Often corresponds to general hospital with teaching function.
- **Tertiary hospital**: often referred to as central, regional or tertiary-level hospital. A hospital ***with highly specialized staff and technical equipment***, e.g., ICU, Haematology, Transplantation, cardio-thoracic surgery, neurosurgery and specialized imaging units; clinical services are highly differentiated by function; provides regional services and regularly takes referrals from other (primary and secondary) hospitals. Often correspond to University hospital.
- **Specialized hospital**: ***Single clinical specialty***, possibly with sub-specialties; highly specialized staff and technical equipment.

### 5.1.2 Participated before?

If your institution has participated before, please login with your existing account. New participants belonging to an institution that has participated before, can be added to this user by the local admin. If the local administrator is no longer working at the institution, or lost their login, please contact [Global-PPS@uantwerpen.be](mailto:Global-PPS@uantwerpen.be).

## **5.2 Prepare the department list**

If you participate for the first time, make sure you add all departments. If your institution has participated before, you need to update the departments (when necessary).

Each department must be entered manually in the Global-PPS tool, with the following information (mandatory variables are marked with *):

- **Unique name of the department***: Enter a name for your department
- **Code and description**: This can help you describe your department in more detail
- **Patient care type***: Choose ‘Outpatients’ for outpatient departments/units
- **Specialty type***: Choose between the following 25 outpatient specialties (choose the most appropriate option):


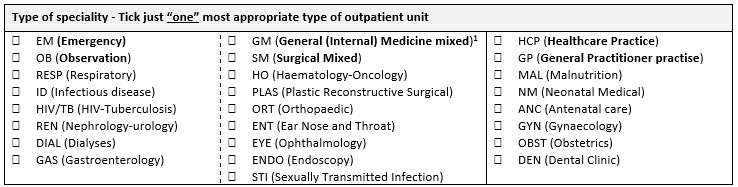


## **5.3 Select your survey period**

Select the appropriate survey period in which you collected your data:

- January-April
- May-August
- September-December

Choose which modules you want to participate in (inpatient, inpatient+HAI module, outpatient).

## **5.4 Enter unit and patient data**

After preparing your departments and choosing a survey period, you can start entering unit and patient data. Make sure each patient is entered in the correct unit.

**Important**: Denominators for the outpatient module are collected by collecting **data for all patients regardless of antimicrobial prescription**. More detailed data is collected for the numerator, i.e. **all patients who receive an antimicrobial prescription**. This is an important difference with the inpatient module!

**Important**: Each unit is linked to the department created in the department list. If the unit specialty type is incorrect, please update your department list to correct this.

## **5.5 Export raw data & download report**

At any time during or after data entry, it is possible to download a Microsoft Excel® file with all raw data. This can help you verify the entered data for missingness & correctness. If you have access to multiple institutions, it is additionally possible to download a merged Excel file, containing raw data of multiple institutions.

**Important**: Each row corresponds to one antimicrobial prescription, or to a patient without any antimicrobial prescriptions. Hence, patients with multiple antimicrobial prescriptions, are presented on multiple rows.

After data entry and finalizing your data entry (please consult our [**§User manuals & Tutorial videos**](file:///N:\FGEN\VAXINFECTIO\Projecten%20Prof%20H.%20Goossens\ESAC\BIOMERIEUX-PPS\protocol-development\2024\Outpatient\Protocol\Protocol%20Global-PPS%20outpatient%20module_Aug2024_clean-layoutupdate2.docx#_User_manuals_&)), you can download one of our pre-analysed reports:

- **One-point feedback report**: containing detailed information of your institution in one survey
- **Merged feedback report**: containing detailed information of >1 institutions in one survey

## **5.6 Technical support**

The Global-PPS Coordinating Centre & Technical Support team at the University of Antwerp provides a help desk for software or any other issues encountered and/or questions during the data collection and data entering ([global-pps@uantwerpen.be](mailto:Global-PPS@uantwerpen.be)). The team is constantly available for general queries about the project.

The Global-PPS tool <https://app.globalpps.uantwerpen.be/globalpps_webpps/> offers:

- ***internal checks*** to avoid invalid or erroneous figures (e.g. for out-of-range values)
- boxes popping up to ***guide you with data entry***
- help functions which provide ***supplementary information*** on each screen
- Help pages, ***User manuals***, ***FAQ page*** (see [**§More questions for us?**](file:///N:\FGEN\VAXINFECTIO\Projecten%20Prof%20H.%20Goossens\ESAC\BIOMERIEUX-PPS\protocol-development\2024\Outpatient\Protocol\Protocol%20Global-PPS%20outpatient%20module_Aug2024_clean-layoutupdate2.docx#_More_questions_for)).

Web page layout for the forms is similar to the paper version. Regular backups of the database will guarantee the integrity of data. The software and database are hosted on a server at the University of Antwerp in Belgium, Europe. The Global-PPS team can provide more details on ensured data protection and safeguarding (contact [global-pps@uantwerpen.be](mailto:Global-PPS@uantwerpen.be)) .

# 6. Getting started

Before conducting the Global-PPS in your institution, you need to organise a multidisciplinary team to help you conduct the survey (see [**§6.1 Organise a multidisciplinary team**](file:///N:\FGEN\VAXINFECTIO\Projecten%20Prof%20H.%20Goossens\ESAC\BIOMERIEUX-PPS\protocol-development\2024\Outpatient\Protocol\Protocol%20Global-PPS%20outpatient%20module_Aug2024_clean-layoutupdate2.docx#_6.1_Organise_a)). In some settings, you need clearance from an ethical committee to conduct the survey (see [**§6.2 Ethical approval**](file:///N:\FGEN\VAXINFECTIO\Projecten%20Prof%20H.%20Goossens\ESAC\BIOMERIEUX-PPS\protocol-development\2024\Outpatient\Protocol\Protocol%20Global-PPS%20outpatient%20module_Aug2024_clean-layoutupdate2.docx#_6.2_Ethical_approval)).

## **6.1 Organise a multidisciplinary team**

### 6.1.1 Participate for the first time?

The healthcare facilities are invited to create a ***multidisciplinary team of colleagues familiar with reading patient notes and having adequate knowledge on local guidelines***.

A ***local administrator*** has to be assigned and he/she will be the main contact person for the Global-PPS Coordinating Centre & Technical Support team at the University of Antwerp, Belgium. The local administrator is responsible for:

- the online registration of the institution (hospital or healthcare facility)
- entering patient-specific data into the Global-PPS tool
- the data validation
- the production of the local feedback reports

Extra hospital users may, however, be registered within the Global-PPS tool in order to help the local administrator with data entry (see our user manuals, [**§User manuals & Tutorial videos**](file:///N:\FGEN\VAXINFECTIO\Projecten%20Prof%20H.%20Goossens\ESAC\BIOMERIEUX-PPS\protocol-development\2024\Outpatient\Protocol\Protocol%20Global-PPS%20outpatient%20module_Aug2024_clean-layoutupdate2.docx#_User_manuals_&)).

### 6.1.2 Participated before?

Please involve your previously established ***multidisciplinary team*** when conducting this survey. Moreover, you need to get in touch with the ***already existing local administrator*** for your institution. If the local administrator is not known or you do not know whether the institution participated in the Global-PPS before, please get in touch with [global-pps@uantwerpen.be](mailto:global-pps@uantwerpen.be).

**Important**: Enter your outpatient antimicrobial prescribing data to the **already existing database for your institution**, because this is the only way to get access to all previously entered data for your institution, which will allow you to retrieve longitudinal feedback reports, including previously entered data. In this way, all antimicrobial use data will be entered in one single database for your institution including both inpatient and outpatient data.

## **6.2 Ethical approval**

For approval by ethical committee & privacy legislation requirements, the Global-PPS Coordinating Centre & Technical Support team can provide, on request, a data privacy excerpt that can be submitted to institutions ethical committees if needed. Further, depending on the local setting and mode of data collection, a patient informed consent form or other common form may be required. Please note that the aim is to collect complete data within the defined timeframe of at least 4 hours (see [**§3.2.2 Timeslot of surveillance**](file:///N:\FGEN\VAXINFECTIO\Projecten%20Prof%20H.%20Goossens\ESAC\BIOMERIEUX-PPS\protocol-development\2024\Outpatient\Protocol\Protocol%20Global-PPS%20outpatient%20module_Aug2024_clean-layoutupdate2.docx#_3.2.2_Timeslot_of)), sample-based data collection should be avoided. For more information contact [global-pps@uantwerpen.be](mailto:Global-PPS@uantwerpen.be).

# 7. Data Management, Privacy & Publication

Please read below our additional information about data privacy ([**§7.1 Data privacy**](file:///N:\FGEN\VAXINFECTIO\Projecten%20Prof%20H.%20Goossens\ESAC\BIOMERIEUX-PPS\protocol-development\2024\Outpatient\Protocol\Protocol%20Global-PPS%20outpatient%20module_Aug2024_clean-layoutupdate2.docx#_7.1_Data_privacy)), data ownership ([**§7.2 Data ownership**](file:///N:\FGEN\VAXINFECTIO\Projecten%20Prof%20H.%20Goossens\ESAC\BIOMERIEUX-PPS\protocol-development\2024\Outpatient\Protocol\Protocol%20Global-PPS%20outpatient%20module_Aug2024_clean-layoutupdate2.docx#_7.2_Data_ownership)) and our publication policy ([**§7.3 Publication policy**](file:///N:\FGEN\VAXINFECTIO\Projecten%20Prof%20H.%20Goossens\ESAC\BIOMERIEUX-PPS\protocol-development\2024\Outpatient\Protocol\Protocol%20Global-PPS%20outpatient%20module_Aug2024_clean-layoutupdate2.docx#_7.3_Publication_policy)).

## **7.1 Data privacy**

A ***sequence number*** is assigned to each institution (hospital or healthcare facility) after registration in the Global-PPS tool. Institution names will never be revealed in any report or publication without approval from the participant (e.g. for peer-reviewed articles).

***Patients are completely pseudonymized*** in the Global-PPS tool. Every patient record will be given a unique non-identifiable survey number. This number is automatically generated by the software application, based on several internal codes. This number identifies the patient uniquely in the Global-PPS database. For more information, consult the data privacy excerpt (contact [global-pps@uantwerpen.be](mailto:global-pps@uantwerpen.be)).

## **7.2 Data ownership**

Data are the ***property of the respective institution***. Entered inpatient and/or outpatient data remain available to the institution at all times.

The Global-PPS Coordinating Centre & Technical Support team at the University of Antwerp, Belgium is ***guardian of the data within the database***;

- They will analyse the data and program the automatic reports. These analyses and reports are property of the Global-PPS;
- They facilitate country- and/or region-specific analyses.

For more information, consult the data privacy excerpt (contact [global-pps@uantwerpen.be](mailto:global-pps@uantwerpen.be)).

## **7.3 Publication policy**

The Global-PPS Coordinating Centre & Technical Support team looks for opportunities for dissemination and encourages country-specific analyses. For publications at national or regional level, participants need to comply with the publication strategy as designed by the Global-PPS Coordinating Centre & Technical Support team. The publication strategy will guide you on how to proceed. The publication policy is available at [www.global-pps.com/documents](http://www.global-pps.com/documents).

# More questions for us?

If you have any more questions for us, please take a look at the following things:

1. Questions about the **tool**, e.g. how to register, login, enter data, or download your report? Please take a look at [**§User manuals & Tutorial videos**](file:///N:\FGEN\VAXINFECTIO\Projecten%20Prof%20H.%20Goossens\ESAC\BIOMERIEUX-PPS\protocol-development\2024\Outpatient\Protocol\Protocol%20Global-PPS%20outpatient%20module_Aug2024_clean-layoutupdate2.docx#_4.1_User_manuals).
2. Questions about the **methodology** and how to **get started**? Please consult our ***Frequently Asked Questions*** (<https://www.global-pps.com/faq/>) list or our ***documents*** (<https://www.global-pps.com/documents/>).

If your answer is not there, please contact us at [global-pps@uantwerpen.be](mailto:global-pps@uantwerpen.be)

1. Questions about our **publication policy**, **ethical approval**, etc.? Please take a look at [**§6.2 Ethical approval**](file:///N:\FGEN\VAXINFECTIO\Projecten%20Prof%20H.%20Goossens\ESAC\BIOMERIEUX-PPS\protocol-development\2024\Outpatient\Protocol\Protocol%20Global-PPS%20outpatient%20module_Aug2024_clean-layoutupdate2.docx#_6.2_Ethical_approval) and [**§7.3 Publication policy**](file:///N:\FGEN\VAXINFECTIO\Projecten%20Prof%20H.%20Goossens\ESAC\BIOMERIEUX-PPS\protocol-development\2024\Outpatient\Protocol\Protocol%20Global-PPS%20outpatient%20module_Aug2024_clean-layoutupdate2.docx#_7.3_Publication_policy). Still have questions? Please contact us [global-pps@uantwerpen.be](mailto:global-pps@uantwerpen.be)

## **User manuals & Tutorial videos**

If you have any questions about data entry, please consult our user manuals and tutorial videos! Go to “**Instructions**” and:

- “**User manual**” to take a look at our user manuals for the Inpatient and Outpatient modules
- “**Tutorial videos**” to watch short instruction videos on registering, logging in, adding users and all details for data entry.


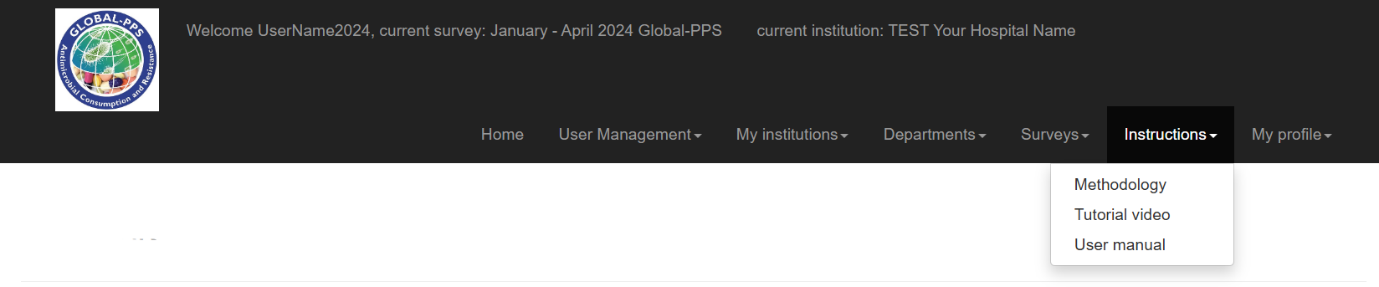


1. Sullivan TJ, Wedner HJ, Shatz GS, Yecies LD, Parker CW. Skin testing to detect penicillin allergy. J Allergy Clin Immunol. 1981 Sep;68(3):171-80. doi: 10.1016/0091-6749(81)90180-9. PMID: 6267115. [↑](#footnote-ref-1)
2. Versporten A, Zarb P, Caniaux I, Gros MF, et al. Antimicrobial consumption and resistance in adult hospital inpatients in 53 countries: results of an internet-based global point prevalence survey. Lancet Glob Health. 2018;**6**:e619-e629. [↑](#footnote-ref-2)
3. WHO’s Anatomical Therapeutic Chemical (ATC) Classification. <https://atcddd.fhi.no/atc_ddd_index/> [↑](#footnote-ref-3)
